# Clinical Decision Support and Cardiometabolic Medication Adherence: A Randomized Clinical Trial

**DOI:** 10.1001/jamanetworkopen.2024.53745

**Published:** 2025-01-09

**Authors:** Patrick J. O’Connor, Jacob L. Haapala, Steven P. Dehmer, Lilian N. Chumba, Heidi L. Ekstrom, Stephen E. Asche, Dan J. Rehrauer, Melissa A. Pankonin, Pamala A. Pawloski, Marsha Raebel, JoAnn M. Sperl-Hillen

**Affiliations:** 1 HealthPartners Institute, Bloomington, Minnesota; 2 HealthPartners, Bloomington, Minnesota; 3 Institute for Health Research, Kaiser Permanente Colorado, Aurora

## Abstract

**Importance:**

Medication adherence is important for managing blood pressure (BP), low-density lipoprotein cholesterol (LDL-C), and hemoglobin A_1c_ (HbA_1c_). Interventions to improve medication adherence are needed.

**Objective:**

To examine the effectiveness of an intervention using algorithmic identification of low medication adherence, clinical decision support to physicians, and pharmacist outreach to patients to improve cardiometabolic medication adherence and BP, LDL-C, and HbA_1c_ control.

**Design, Setting, and Participants:**

A 2-arm, patient-randomized, parallel group clinical trial was conducted. Twenty-six primary care clinics using effective decision support to encourage timely adjustments of cardiometabolic medications were included. On the date of an index visit, participants were (1) aged 18 to 75 years, (2) receiving a statin or not at the goal level for HbA_1c_ or BP, and (3) had proportion of days covered less than 80% for 1 or more BP or noninsulin glucose-lowering medications or a statin. The study was conducted from August 19, 2020, to September 30, 2023. Data analysis was performed from October 1, 2023, to August 30, 2024.

**Intervention:**

Electronic health record–linked clinical decision support identified and encouraged discussion of medication adherence issues. For patients in the intervention cohort continuing to meet eligibility criteria 6 months after an index visit, pharmacist telephone outreach was attempted.

**Main Outcomes and Measures:**

The main outcomes of the trial were (1) adherence to selected classes of cardiometabolic medications, (2) control of HbA_1c_, BP, or LDL-C levels at 12 months after the index visit, and (3) costs of care.

**Results:**

Among 5421 participants (2990 [55%] male; mean [SD] age, 57 [11] years) 12 months after the index date, intervention patients had better adherence to BP medications (adjusted odds ratio [AOR], 1.29; 95% CI, 1.06-1.56), but no better adherence to statins (AOR, 1.18; 95% CI, 0.99-1.41) or noninsulin diabetes medications (AOR, 1.03; 95% CI, 0.82-1.30) compared with patients receiving usual care. The intervention did not improve mean HbA_1c_ (−0.2%; 95% CI, −0.4 to 0.1), systolic BP (1.4 mm Hg; 95% CI, −0.8 to 3.5 mm Hg), or LDL-C (−1.8 mg/dL; 95% CI, −6.5 to 2.8 mg/dL). Compared with usual care, intervention patients eligible for pharmacist outreach had improved HbA_1c_ (−0.4%; 95% CI, −0.8% to −0.1%) compared with those not eligible for outreach (−0.0; 95% CI, −0.3% to 0.3%). Health care use costs did not differ significantly between study arms.

**Conclusions and Relevance:**

This cost-neutral intervention increased adherence to BP medications, but not to statins or glucose-lowering medications, with no overall improvement in BP, LDL-C, or HbA_1c_ control. Modifications of this intervention strategy are needed to improve cardiometabolic risk factor control.

**Trial Registration:**

ClinicalTrials.gov Identifier: NCT03748420

## Key Points

**Question:**

Does algorithmic identification of low medication adherence plus corresponding clinical decision support to primary care clinicians, combined with targeted pharmacist outreach, improve cardiometabolic medication adherence and blood pressure, cholesterol, or glucose level control?

**Findings:**

In this randomized clinical trial of 5421 adults, the intervention improved blood pressure adherence. However, statin adherence, glucose medication adherence, overall blood pressure, cholesterol, and glucose level control were not improved.

**Meaning:**

The findings of this trial suggest that algorithmic identification of medication nonadherence in primary care with decision support to primary care clinicians and targeted pharmacist outreach is feasible and may improve adherence to some cardiometabolic medications.

## Teaser

This randomized clinical trial examines the use of algorithmic identification of nonadherence, provision of clinical decision support to primary care clinicians, and pharmacist outreach to patients to improve adherence to medications treating hypertension, diabetes, and dyslipidemia.

## Introduction

Suboptimal medication adherence is common in adults treated for type 2 diabetes, hypertension, or dyslipidemia, and low medication adherence is widely recognized as a major barrier to better clinical outcomes in individuals with cardiometabolic conditions.^1,2,3,4,5,6,7,8,9^ However, it is difficult for clinicians to accurately identify low medication adherence, in part because patients may not disclose accurate adherence information for various reasons.

Informatics advances now merge medication prescription and dispensing data, enabling near real-time estimation of an individual patient’s adherence to prescription medications by comparing the number of days of medication supplied with the number of calendar days elapsed between medication fills. However, primary care clinician (PCC) access to adherence data currently requires multiple interruptive mouse clicks and is infrequently performed.

In an effort to improve patient adherence to diabetes, hypertension, and dyslipidemia medications, we designed an intervention that communicates drug-specific medication adherence data directly to PCCs and patients at primary care clinical encounters and provides subsequent proactive pharmacist outreach to patients with persistent low adherence and suboptimal diabetes, hypertension, or dyslipidemia control 6 months after the patient’s index visit. Outreach community and health plan pharmacists were trained to identify and address factors that may contribute to low medication adherence and associated poor control of chronic conditions,^10^ using an approach endorsed by the World Health Organization.^1^ We hypothesized that an intervention designed to identify and address medication nonadherence would improve not only medication adherence, but also contribute to better diabetes, hypertension, and dyslipidemia control.

## Methods

### Study Design, Setting, and Participants

This patient-randomized clinical trial was conducted at an integrated health care system in the upper Midwest. Participant accrual began on August 19, 2020, and continued for 6 months, and outcomes were assessed during a patient-specific 18-month postindex follow-up period. The trial was completed on September 30, 2023. The trial protocol is presented in Supplement 1. This study was reviewed in advance, approved, and monitored by the HealthPartners Institutional Review Board, and overseen by a National Institutes of Health–approved data safety and monitoring board. The institutional review board granted a waiver of written informed consent for patients based on minimal risk. This study followed the Consolidated Standards of Reporting Trials (CONSORT) reporting guideline.

Patients were eligible for the study if they were aged 18 to 75 years, received care at 1 of 26 primary care clinics, and met criteria for 1 or more of 3 study cohorts (not mutually exclusive) on the date of an index primary care clinical encounter. The first cohort comprised individuals undergoing treatment for hypertension, with blood pressure (BP) greater than or equal to 140/90 mm Hg and proportion of days covered (PDC) less than 80% for all prescribed medications in at least 1 hypertension medication class (thiazide or loop diuretics, angiotensin-converting enzyme inhibitor, angiotensin receptor blocker, calcium channel blocker, or β-blocker).

The diabetes cohort included individuals with hemoglobin A_1c_ (HbA_1c_) greater than or equal to 8% (to convert to proportion of total hemoglobin, multiply by 0.01) and PDC less than 80% for all medications in at least 1 of the noninsulin glucose-lowering medication classes (metformin, sulfonylurea, meglitinide, glucagonlike peptide-1 receptor agonist, sodium-glucose cotransporter 2 inhibitor, dipeptidyl peptidase-4 inhibitor, or thiazolidinedione).

Participants in the dyslipidemia cohort met American College of Cardiology/American Heart Association (ACC/AHA) criteria for statin use and receiving a statin with PDC less than 80%. Patients with their most recent low-density lipoprotein cholesterol (LDL-C) less than 100 mg/dL (to convert to millimoles per liter, multiply by 0.0259) within 24 months before the index encounter were excluded. The ACC/AHA criteria for statin use included the following. First, age greater than 21 years and either (1) LDL-C greater than or equal to 190 mg/dL, (2) atherosclerotic cardiovascular disease (ASCVD) diagnosis on the problem list; or (3) 2 or more *International Statistical Classification of Diseases and Related Health Problems, 10th Revision* (*IDC-10*)^11^ ASCVD encounter codes in the previous 2 years. Second, age 40 to 75 years and either (1) diagnosis of diabetes based the problem list or 2 or more *IDC-10* encounter codes in the previous 2 years or (2) a 10-year ASCVD risk score of 7.5% or more based on the ACC/AHA 10-year pooled cohort equations.^12^

Pregnant patients and those living in long-term care facilities or receiving parenteral cancer chemotherapy or hospice care were excluded from the study. Patients opting out of research were excluded from the analysis.

### Randomization

Patient-level randomization of study-eligible patients was done at the time of an index primary care encounter at a study clinic during a 6-month accrual period. All patients meeting study eligibility criteria at an index visit were assigned a random 7-digit study identification number by a web-based computerized allocation system. Patients were allocated to either clinical decision support or usual care based on the random even or odd terminal digit of the study identification number.

### Intervention

The intervention was implemented from August 19, 2020, to August 19, 2022, and was designed with end-user input to (1) provide medication-specific adherence information for cardiometabolic medication to PCCs and study-eligible patients at the index and subsequent primary care encounters, (2) establish and maintain a registry of study-enrolled patients eligible for proactive pharmacist outreach, and (3) standardize the pharmacist outreach strategy based on the World Health Organization–endorsed^1^ information-motivation-behavioral skills (IMB) model for medication adherence. This model proposes that medication adherence is primarily due to behavior, and informed patients who are highly motivated and possess the skills for medication adherence have a greater likelihood of exhibiting appropriate and consistent medication-taking behaviors across disease states and populations. The IMB model has been shown to support variation in medication adherence in up to half of patients studied.^13,14,15,16^

Clinic rooming staff were prompted to print the clinical decision support output at the start of clinical encounters and to hand the PCC version to the clinician and the patient version to the patient for separate review before the PCC entering the examination room. An additional description of clinical decision support design and function is provided elsewhere.^17,18,19,20,21,22,23,24,25^

Clinical decision support intervention patients were reassessed 6 months after the index visit, and those with both persistent low medication adherence and persistent above-recommended care goals were placed in a registry for proactive pharmacist outreach. Community-based and health plan–employed pharmacists used the IMB framework and scripted templates with patients to develop individualized medication adherence plans that could include education about specific medications and their adverse effects, strategies to reduce medication costs, simplifying the refill process, or using reminder systems or pill boxes. Pharmacists had EHR access for documentation and could make medication changes by using established care protocols or communicating with prescribers. Pharmacists were trained in the use of the IMB framework, but were not trained to recognize or manage depression.

### Data Collection

The EHR data, including vital signs, diagnosis and procedure codes, medication prescriptions, EHR-based PDC values, laboratory data, medication allergies, and demographic information were stored in a firewall-protected analytic database for analysis. Health care costs comprised use of clinic-based outpatient services, including primary care, urgent care, specialty care, and laboratory services but excluding emergency, same-day surgery, and inpatient services, and pharmacy fills. Costs related to the clinical decision support intervention, including the pharmacist outreach, were also excluded. Medical procedure and medication codes were converted to 2021 US dollars using total care relative resource values, a nationally representative and standardized set of pricing measures derived from Centers for Medicare & Medicaid Services relative value units.^26^

### Outcomes

Coprimary outcomes were defined for each study cohort and measured using data from the EHR collected in the course of clinical care. A detailed description of how medication adherence was estimated is included in the eMethods in Supplement 2. Predefined secondary outcomes included intervention impact at 18-month follow-up, heterogeneity of intervention effects, and intervention effect on health care use costs.

#### Hypertension Cohort

The adherence outcome in the hypertension cohort was achievement of PDC greater than or equal to 80% for at least 1 antihypertensive medication in each currently active BP medication class 12 months after the index date. The clinical outcome was change from the last recorded systolic BP (SBP) at the index encounter to the last recorded SBP at the encounter closest to the date 12 months after the index date. Clinic staff were trained to use digital BP equipment, and the recorded BP is typically the average of the last 2 of 3 BP measures at the clinical encounter.

#### Diabetes Cohort

The adherence outcome in the diabetes cohort was achievement of PDC greater than or equal to 80% for at least 1 medication in each currently prescribed noninsulin glycemic medication class 12 months after the index date. The clinical outcome was change in HbA_1c_ from most recent HbA_1c_ on or before the index date to the subsequent HbA_1c_ closest to the 12-month follow-up date.

#### Dyslipidemia Cohort

The adherence outcome in the dyslipidemia cohort was achievement of a PDC greater than or equal to 80% for a statin if currently prescribed at 12 months following the index office visit. The clinical outcome was change in the LDL-C from the index date to the LDL-C closest to the 12-month follow-up date. This outcome was promoted from secondary to primary outcome before analysis of results.

### Adherence Assessment

Electronic health record–derived (Epic Systems) PDC measures were computed by comparing each patient’s then-current active outpatient medications with the number of times a medication had been dispensed, including the number of days supplied, based on a 6-month observation period before the index visit date for each patient to determine study eligibility. The PDC was calculated in the same way at 6, 12, and 18 months after the index date to determine eligibility for pharmacist outreach at 6 months and assess medication adherence outcomes at 12 and 18 months after the index date. The EHR-derived PDC measure included a confidence score calculated for each medication adherence calculation based on the reliability of the source data. For example, medications with dispense history obtained from pharmacy records are generally regarded with higher confidence than medication dispense records received from administrative data (moderate) or recorded samples (low).

Adherence was assessed stepwise as follows. First, a list of active drugs within each drug class was assembled on the date of interest. Examples of drug classes include statins, biguanide, sulfonylureas, thiazide diuretics, angiotensin-converting enzyme inhibitors, and angiotensin receptor blockers. Second, for patients to be classified as adherent at that time to each drug class with 1 or more active drug, at least 1 of the active drugs within that drug class had to have a PDC greater than or equal to 80%. Third, if the patient did not meet the adherent classification, they were classified as nonadherent to that drug class. Fourth, if on the date of interest the patient was receiving more than 1 (for example, BP) drug class, then the patient was required to be adherent to all BP drug classes active on the date of interest to be classified as adherent to BP medications on that date. Fifth, the same rules were applied separately for BP, glucose-lowering, and statin medications. Additional details on how medication adherence was calculated is provided in the eMethods in Supplement 2.

### Statistical Analysis

Data analysis was performed from October 1, 2023, to August 30, 2024. The expected numbers of study-eligible patients by cohort were 1066 (hypertension), 832 (diabetes), and 1694 (dyslipidemia) in a 6-month accrual period. Given these sample sizes, at α = .05, we estimated greater than or equal to 80% a priori power to detect absolute differences in proportions by study arm of patients becoming adherent to medications at 12 months of greater than or equal to 9.9% (diabetes), greater than or equal to 8.7% (hypertension), and greater than or equal to 6.9% (dyslipidemia). Additionally, under similar assumptions, we estimated greater than or equal to 80% power to detect differences of greater than or equal to 3 mm Hg in mean SBP and greater than or equal to 0.3% in mean HbA_1c_ by study group. With 1 or 2 principal outcomes for each cohort, we did not adjust for multiple comparisons.

We used generalized linear regression models for adherence end points and general linear mixed regression models with a random patient intercept for clinical end points to estimate the effect of the intervention. Models for adherence at 12 months featured a binary dependent variable for adherence to all currently prescribed medication classes defined as a PDC greater than or equal to 80% for at least 1 medication in each currently prescribed medication class, a binary study arm predictor, and baseline covariates selected a priori of age, sex, and race and ethnicity (based on patient self-report in the EHR) to potentially improve the efficiency of the analysis. Estimates from these models are reported as adjusted odds ratios (AORs) (binomial distribution, logit link function) comparing the probability of adherence in clinical decision support intervention patients vs usual care patients, along with 95% CIs. Models for SBP, LDL-C, and HbA_1c_ had continuous dependent variables, a binary study arm predictor, time (index vs 12 months), and a treatment by time interaction term to assess treatment effect. Estimates from these models are reported as β estimates (difference in means by group, normal distribution, identity link function) with 95% CIs. Analysis was performed using SAS, version 9.4 (SAS Institute Inc).

Exploratory analyses evaluated heterogeneity of treatment effects by eligibility for pharmacist outreach, patient sex, age group, race and ethnicity, and insurance class by including interaction terms in the models. Other heterogeneity effects evaluated included patient interpreter needs, presence of comorbidities (ie, depression, cardiovascular disease, and chronic kidney disease), potential number of exposures at postindex encounters, and baseline levels of SBP, LDL-C, and HbA_1c_. Tests were 2-sided, and *P* < .05 was considered significant for these exploratory analyses.

Health care use costs were modeled using generalized estimating equations with a binary study arm predictor, time (12 pre vs post index), a treatment by time interaction term, baseline covariates of age, sex, race and ethnicity, and health insurance status, and a random patient intercept. A planned cost-effectiveness analysis was not done due to lack of intervention effect on clinical outcomes.

## Results

**Figure 1.**
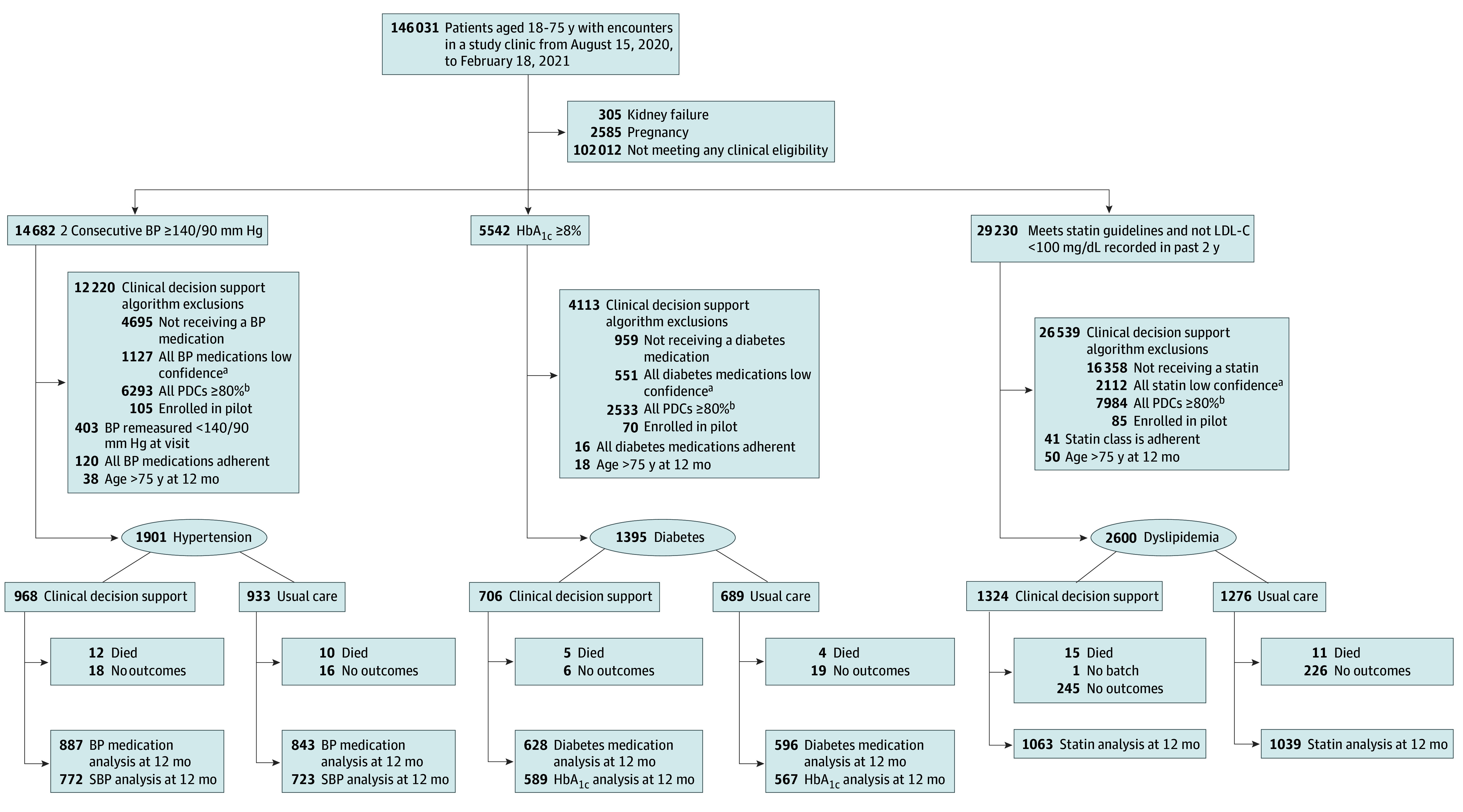
Eligibility, Randomization, and Inclusion in Primary End Point Analysis BP indicates blood pressure; HbA_1c_, hemoglobin A_1c_; and PDC, proportion of days covered; and SBP, systolic BP. ^a^Proportion of days covered estimates are low confidence if dispensed data are uncertain, as defined in the eMethods in Supplement 2. ^b^PDC indicates proportion of days covered.

The CONSORT diagram (Figure 1) shows how study eligibility criteria were applied to identify 3 study-eligible cohorts. Table 1 reports the baseline characteristics of the 5421 patients who had an index visit during the 6-month accrual period and were eligible for inclusion in the analysis. The population included 2990 men (55%) and 2431 women (45%); mean (SD) age was 57 (11) years. An individual patient may have been eligible for more than one study cohort, for example, among those in the diabetes cohort, 7% were also in the hypertension cohort and 17% were in the dyslipidemia cohort.

**Table 1.** Baseline Characteristics of Study-Eligible Patients

Characteristic	Patients, No. (%)							
	All		Hypertension cohort		Diabetes cohort		Dyslipidemia cohort	
	Clinical decision support (n=2745)	Usual care (n=2676)	Clinical decision support (n=968)	Usual care (n=933)	Clinical decision support (n=706)	Usual care (n=689)	Clinical decision support (n=1324)	Usual care (n=1276)
Cohort								
Diabetes	706 (26)	689 (26)	52 (5)	40 (4)	706 (100)	689 (100)	126 (10)	116 (9)
Hypertension	968 (35)	933 (35)	968 (100)	933 (100)	52 (7)	40 (6)	91 (7)	83 (7)
Dyslipidemia	1324 (48)	1276 (48)	91 (9)	83 (9)	126 (18)	116 (17)	1324 (100)	1276 (100)
Sex								
Female	1235 (45)	1196 (45)	449 (46)	429 (46)	323 (46)	325 (47)	572 (43)	542 (42)
Male	1510 (55)	1480 (55)	519 (54)	504 (54)	383 (54)	364 (53)	752 (57)	734 (58)
Age, y								
Mean (SD), y	57 (11)	58 (11)	54 (12)	55 (12)	55 (11)	56 (11)	60 (9)	61 (9)
18-44	381 (14)	355 (13)	211 (22)	198 (21)	127 (18)	120 (17)	68 (5)	64 (5)
45-54	656 (24)	578 (22)	259 (27)	238 (26)	189 (27)	167 (24)	281 (21)	236 (18)
55-64	898 (33)	949 (35)	280 (29)	278 (30)	239 (34)	240 (35)	480 (36)	505 (40)
65-74	810 (30)	794 (30)	218 (23)	219 (23)	151 (21)	162 (24)	495 (37)	471 (37)
Race and ethnicity^a^								
Asian	188 (7)	205 (8)	62 (6)	58 (6)	53 (8)	70 (10)	95 (7)	98 (8)
Black	688 (25)	697 (26)	282 (29)	280 (30)	202 (29)	201 (29)	305 (23)	302 (24)
Hispanic	116 (4)	114 (4)	26 (3)	32 (3)	63 (9)	48 (7)	47 (4)	48 (4)
White	1622 (59)	1549 (58)	558 (58)	530 (57)	345 (49)	329 (48)	820 (62)	774 (61)
Other/unknown^b^	131 (5)	111 (4)	40 (4)	33 (4)	43 (6)	41 (6)	57 (4)	54 (4)
Needs interpreter	240 (9)	252 (9)	69 (7)	68 (7)	80 (11)	80 (12)	135 (10)	137 (11)
Insurance								
Medicaid^c^	723 (26)	740 (28)	262 (27)	270 (29)	232 (33)	232 (34)	320 (24)	304 (24)
Medicare	715 (26)	695 (26)	196 (20)	185 (20)	147 (21)	140 (20)	415 (31)	419 (33)
Commercial	1238 (45)	1160 (43)	480 (50)	447 (48)	314 (44)	294 (43)	561 (42)	517 (41)
Other/none	69 (3)	81 (3)	30 (3)	31 (3)	13 (2)	23 (3)	28 (2)	36 (3)
Depression diagnosis	624 (23)	599 (22)	223 (23)	191 (20)	175 (25)	170 (25)	277 (21)	283 (22)
Cardiovascular risk, %^12^^d^								
0 to <7.5	681 (25)	683 (26)	347 (36)	362 (39)	210 (30)	199 (29)	156 (12)	156 (12)
7.5 to <10	275 (10)	288 (11)	84 (9)	71 (8)	44 (6)	53 (8)	168 (13)	182 (14)
10 to <20	815 (30)	757 (28)	218 (23)	189 (20)	158 (22)	161 (23)	502 (38)	453 (36)
≥20	621 (23)	625 (23)	223 (23)	224 (24)	192 (27)	198 (29)	310 (23)	297 (23)
Cardiovascular disease	353 (13)	323 (12)	96 (10)	87 (9)	102 (14)	78 (11)	188 (14)	188 (15)
Diabetes diagnosis	1310 (48)	1254 (47)	266 (27)	216 (23)	702 (99)	681 (99)	552 (42)	533 (42)
HbA_1c_ in patients with diabetes								
Mean (SD), %	8.8 (2.1)	8.6 (2.0)	8.1 (2.1)	7.9 (1.8)	9.9 (1.7)	9.7 (1.6)	8.0 (2.1)	7.8 (1.8)
HbA_1c _≥9	493 (39)	459 (37)	68 (26)	50 (24)	422 (61)	398 (59)	128 (24)	120 (23)
HbA_1c _≥8	812 (64)	776 (63)	102 (40)	73 (35)	694 (100)	677 (100)	200 (38)	185 (36)
SBP, mm Hg								
Mean (SD)	141 (20)	141 (20)	153 (17)	154 (16)	135 (20)	134 (19)	137 (19)	137 (19)
≥150	846 (31)	822 (31)	490 (51)	500 (54)	155 (22)	118 (17)	304 (23)	290 (23)
≥140	1396 (51)	1386 (52)	791 (82)	798 (86)	253 (36)	214 (31)	516 (39)	513 (40)
DBP, mm Hg								
Mean (SD)	85 (14)	85 (13)	93 (13)	94 (12)	81 (12)	81 (11)	82 (12)	82 (12)
≥90	1035 (38)	983 (37)	659 (68)	653 (70)	164 (23)	129 (19)	308 (23)	297 (23)
≥80	1837 (67)	1764 (66)	853 (88)	820 (88)	387 (55)	371 (54)	768 (58)	735 (58)
LDL-C, mg/dL								
Mean (SD)	117 (40)	118 (40)	112 (37)	114 (36)	99 (39)	99 (41)	131 (37)	131 (38)
≥130	866 (34)	838 (34)	263 (32)	255 (33)	142 (21)	135 (20)	568 (43)	543 (43)
≥100	1771 (69)	1728 (70)	507 (61)	505 (65)	295 (43)	286 (43)	1168 (89)	1120 (89)
Patients prescribed the indicated No. of condition-specific medication classes at baseline								
1 Class	1715 (62)	1704 (64)	413 (43)	418 (45)	302 (43)	295 (43)	1324 (100)	1276 (100)
2 Classes	603 (22)	590 (22)	351 (36)	330 (35)	276 (39)	269 (39)	NA	NA
≥3 Classes	427 (16)	382 (14)	204 (21)	185 (20)	128 (18)	125 (18)	NA	NA
Patients with 1 vs ≥2 baseline condition-specific medication classes assessed as low adherence at baseline								
1 Class	2114 (77)	2120 (79)	651 (67)	641 (69)	513 (73)	521 (76)	1324 (100)	1276 (100)
≥2 Classes	631 (23)	556 (21)	317 (33)	292 (31)	193 (27)	168 (24)	NA	NA

Abbreviations: DBP, diastolic blood pressure; HbA_1c_, hemoglobin A_1c_; LDL-C, low-density lipoprotein cholesterol; NA, not applicable; SBP, systolic blood pressure.

SI conversion factors: To convert HbA_1c_ to proportion of total hemoglobin, multiply by 0.01; LDL-C to millimoles per liter, multiply by 0.0259.

^a^ Race and ethnicity categories are based on patient self-report in the electronic health record.

^b^ No further explanation is available for this category.

^c^ Patients with Medicaid plus other insurance are classified as Medicaid.

^d^ Estimated 10-year risk of a nonfatal myocardial infarction, nonfatal stroke, or cardiovascular death based on pooled risk equations.^12^

Table 2 reports that, at 12-month follow-up, intervention patients had significantly better adherence to BP medications than usual care patients (AOR, 1.29; 95% CI, 1.06-1.56), but no significant change in stain adherence (AOR, 1.18; 95% CI, 0.99-1.41) or adherence to noninsulin glucose-lowering medications (AOR, 1.03; 95% CI, 0.82-1.30). Crude and adjusted results were similar.

**Table 2.** Efficacy Outcomes for Medication Adherence and Changes in Clinical Values

Outcome	Study arm, No./total No. (%) or mean (No.)		Clinical decision support vs usual care, OR or β (95% CI)	
	Clinical decision support	Usual care	Unadjusted	Adjusted
**Primary end points**				
Adherence at 12 mo				
BP control medications^a^	470/887 (53.0)	396/843 (47.0)	OR: 1.27 (1.05 to 1.54)	OR: 1.29 (1.06 to 1.56)
Glucose control medications^a^	296/628 (47.1)	279/596 (46.8)	OR: 1.01 (0.81 to 1.27)	OR: 1.03 (0.82 to 1.30)
Statins^b^	586/1063 (55.1)	529/1039 (50.9)	OR: 1.18 (1.00 to 1.41)	OR: 1.18 (0.99 to 1.41)
Change in clinical values at 12 mo, mean (No. of patients)^c^				
SBP, mm Hg	−13.8 (772)	−15.2 (723)	β: 1.4 (−0.8 to 3.5)	β: 1.4 (−0.8 to 3.5)
DBP, mm Hg	−8.2 (772)	−9.4 (723)	β: 1.3 (−0.1 to 2.7)	β: 1.3 (−0.1 to 2.7)
HbA_1c_, %	−1.2 (589)	−1.1 (567)	β: −0.2 (−0.4 to 0.1)	β: −0.2 (−0.4 to 0.1)
LDL-C, mg/dL	−24.7 (612)	−22.9 (587)	β: −1.8 (−6.5 to 2.8)	β: −1.8 (−6.5 to 2.8)
**Additional efficacy end points**				
Adherence at 18 mo				
BP medications	418/831 (50.3)	400/814 (49.1)	OR: 1.05 (0.86 to 1.27)	OR: 1.06 (0.87 to 1.29)
Diabetes medications	228/594 (38.4)	243/574 (42.3)	OR: 0.85 (0.67 to 1.07)	OR: 0.86 (0.68 to 1.08)
Statins	549/1018 (53.9)	502/1017 (49.4)	OR: 1.20 (1.01 to 1.43)	OR: 1.20 (1.00 to 1.44)
Change in clinical values at 18 mo, mean (No.)^c^				
SBP, mm Hg	−13.8 (815)	−14.4 (780)	β: 0.6 (−1.4 to 2.6)	β: 0.6 (−1.4 to 2.6)
DBP, mm Hg	−8.4 (815)	−9.4 (780)	β: 1.0 (−0.2 to 2.3)	β: 1.0 (−0.2 to 2.3)
HbA_1c_, %	−1.1 (604)	−1.1 (582)	β: −0.0 (−0.3 to 0.2)	β: −0.0 (−0.3 to 0.2)
LDL-C, mg/dL	−21.8 (844)	−19.5 (863)	β: −2.3 (−6.0 to 1.4)	β: −2.3 (−6.0 to 1.4)
Change in medical costs at 12 mo, mean (No.), $^c^				
Clinic-based costs	1203 (2745)	1210 (2676)	β: −7 (−126 to $111)	β: −7 (−125 to 110)
Pharmacy costs	1435 (2745)	1273 (2676)	β: 162 (−110 to 434)	β: 162 (−110 to 433)
Pharmacy costs for BP control, glucose control, and statin medications, $	948 (2745)	917 (2676)	β: 31 (−156 to 219)	β: 28 (−159 to 214)

Abbreviations: BP, blood pressure; DBP, diastolic blood pressure; HbA_1c_, hemoglobin A_1c_; LDL-C, low-density lipoprotein cholesterol; OR, odds ratio; SBP, systolic BP.

SI conversion factors: to convert HbA_1c_ to proportion of total hemoglobin, multiply by 0.01; LDL-C to millimoles per liter, multiply by 0.0259.

^a^ Proportion of days covered greater than or equal to 80% for at least 1 medication in each currently prescribed antihypertensive or noninsulin glycemic medication class at 12 months following the index office visit.

^b^ 80% Proportion of days covered greater than or equal to 80% for a statin medication if currently prescribed at 12 months following the index office visit.

^c^ Mean changes listed are unadjusted.

**Figure 2.**
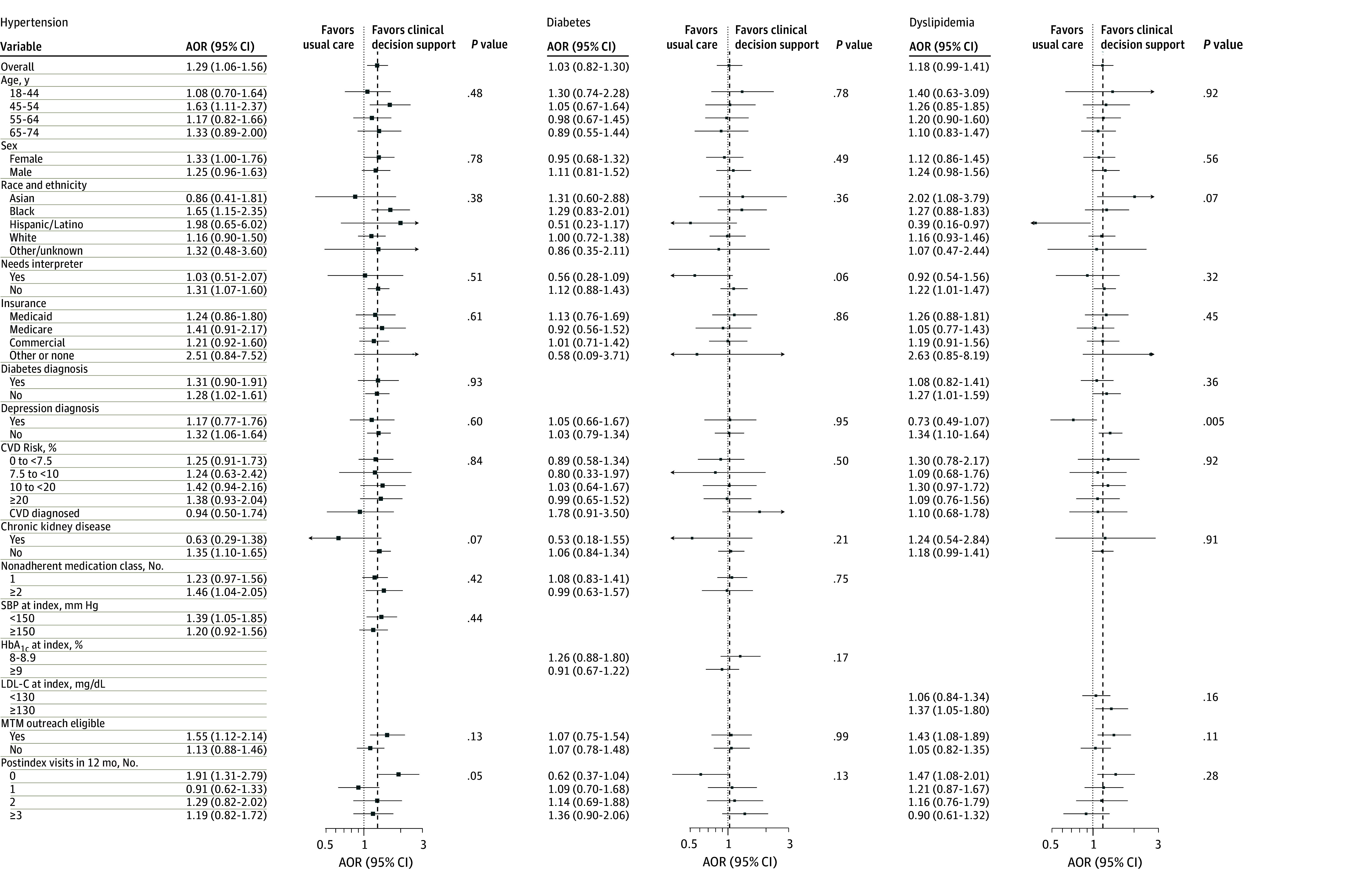
Treatment Effect Heterogeneity for Odds of Medication Adherence at 12 Months Postindex AOR indicates adjusted odds ratio; CVD, cardiovascular disease; HbA_1c_, hemoglobin A_1c_; MTM, medication therapy management; and SBP, systolic blood pressure. SI conversion factors: To convert HbA_1c_ to proportion of total hemoglobin, multiply by 0.01; LDL-C to millimoles per liter, multiply by 0.0259.

Figure 2 shows heterogeneity of the intervention effects on 12-month adherence outcomes for defined subgroups of patients. Of 2745 intervention patients, 1100 (40.1%) were eligible for pharmacist outreach at 6 months. Of these patients, 485 (44.1%) had outreach completed, 250 (22.7%) were deemed ineligible on further review by the pharmacist so that no attempt at outreach was made, and 365 (33.2%) could not be reached or declined. Compared with patients not eligible for pharmacist outreach, the entire group of those eligible for outreach had a directionally stronger and statistically significant intervention effect on medication adherence for those in the hypertension cohort (AOR, 1.55; 95% CI, 1.12-2.14) and the dyslipidemia cohort (AOR, 1.43; 95% CI, 1.08-1.89), but not the diabetes cohort (AOR, 1.07; 95% CI, 0.75-1.54). The intervention effect on statin adherence was greater in those without vs with depression (AOR, 1.34; 95% CI, 1.10-1.54; *P* = .005 for interaction).

There was no significant intervention effect on mean change from the index date to 12 months for SBP (1.4 mm Hg; 95% CI, −0.8 to 3.5 mm Hg), HbA_1c_ (−0.2%; 95% CI, −0.4% to 0.1%), or LDL-C (−1.8 mg/dL; 95% CI, −6.5 to 2.8 mg/dL) (Table 2). Similar results were observed at the 18-month follow-up. There was no significant intervention effect on mean change from 12 months before the index date to 12 months after for clinic-based medical costs (−$7; 95% CI, (−$125 to $110), pharmacy costs overall ($162; 95% CI, −$110 to $433), or pharmacy costs for hypertension, statin medications, or diabetes medications ($28; 95% CI, −$159 to $214).

To assess the extent of clustering, we examined intraclass correlation coefficients (ICCs) at the PCC and clinic level for the primary end points. Primary care clinician–level ICCs ranged from 0.0001 to 0.016, and clinic-level ICCs ranged from 0.0001 to 0.012, suggesting no meaningful level of clustering at these levels.

**Figure 3.**
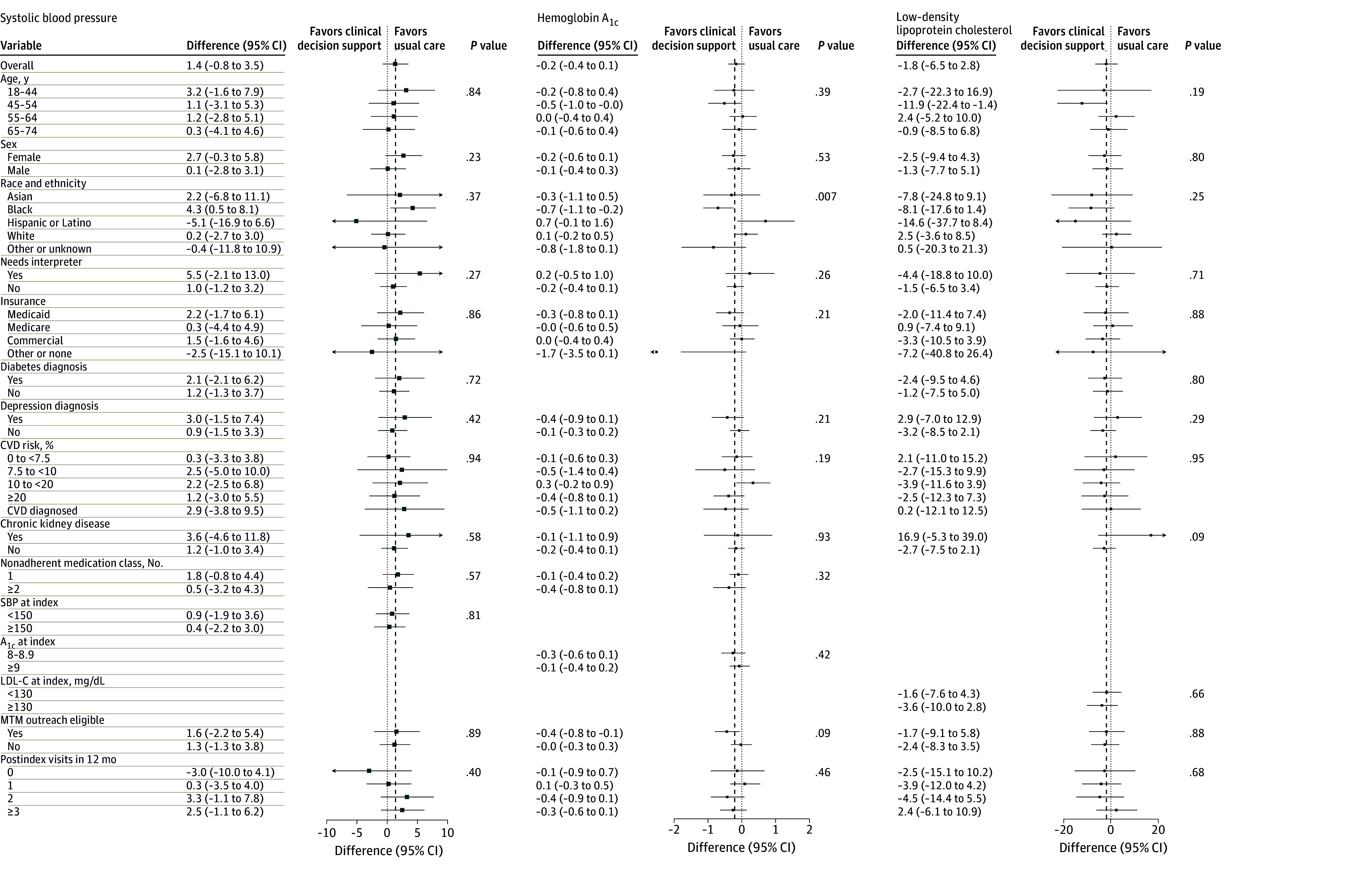
Treatment Effect Heterogeneity for Mean Change in Clinical End Points at 12 Months Postindex CVD, cardiovascular disease; HbA_1c_, hemoglobin A_1c_; MTM, medication therapy management; and SBP, systolic blood pressure. SI conversion factors: To convert HbA_1c_ to proportion of total hemoglobin, multiply by 0.01; LDL-C to millimoles per liter, multiply by 0.0259.

Figure 3 presents exploratory analysis of heterogeneity of intervention effects on change in SBP, LDL-C, and HbA_1c_ from the index date to 12-month follow-up. Black patients had a significant improvement in HbA_1c_ levels with the intervention (−0.7; 95% CI, −1.1 to −0.2; *P* = .007 for interaction). Those eligible for pharmacist outreach at 6 months after the index visit had a directionally larger improvement in HbA_1c_ levels in the intervention group (−0.4%; 95% CI, −0.8% to −0.1%) than those not eligible for pharmacist outreach (−0.0; 95% CI, −0.3% to 0.3%). For BP and LDL-C control, intervention effects did not differ significantly based on eligibility for pharmacist outreach.

The data safety and monitoring board analyses compared patients in the clinical decision support and usual care cohorts and showed no adverse safety signals for diagnosis-defined rates of hypoglycemia, hypotension, hypokalemia, hyperkalemia, hyponatremia, kidney dysfunction, overall hospitalizations, emergency department visits, or deaths.

## Discussion

The clinical decision support intervention led to a clinically and statistically significant 29% increase in adherence to BP medications and a nonsignificant 18% change in statin adherence at 12 months, while not improving adherence to noninsulin glucose-lowering medications. The intervention impact was greatest in those who were eligible for pharmacist outreach at 6 months after the index date, with a 55% decrease in nonadherence to BP medications and a 43% decrease in statin nonadherence in that group (Figure 2). These outcomes were not associated with increases in clinic-based medical care or pharmacy costs for patients receiving the intervention.

These mixed results on medication adherence did not translate to overall better BP, LDL-C, or HbA_1c_ control. The observed benefits of pharmacist involvement suggest that future studies might consider more broadly targeted or more intensive pharmacist outreach.^10^ Outreach pharmacists were able to engage only about half of those who were eligible, but the overall group of patients eligible for pharmacist outreach had significantly less nonadherence to statins and BP medications and significantly better HbA_1c_ levels 12 months after the index date compared with the corresponding patients in the usual care group classified as outreach eligible. These findings suggest that active pharmacist outreach to patients with persistent nonadherence not at clinical goals may be considered to improve care of patients with these common chronic conditions. Efforts to improve adherence may not lead to better control of BP, LDL-C, or HbA_1c_ if the medication regimen is not intensive enough to achieve care goals.^27,28^ This set of issues deserves further exploration, and developing integrated interventions that jointly address medication adherence and clinical inertia may be warranted.^29^

Primary care clinician reception of the medication-specific adherence information was mixed. Some patients identified as having low PDC based on EHR data claimed to be adherent, leading some PCCs to conclude that the PDC data were incorrect. Also, despite baseline training, some PCCs in the clinical decision support group failed to notice the displayed adherence information, which led us to modify how medication-specific adherence information was displayed to PCCs and conduct additional PCC training 6 months after the intervention initiation date. The presentation of PDC data is illustrated in an earlier publication.^30^

Exploratory analysis suggested that the intervention may have improved HbA_1c_ levels in Black individuals and may have been less effective for some adherence outcomes in patients with depression. Prior studies suggest that active comanagement of diabetes and depression may be beneficial, but depression management was not addressed by the study intervnetion.^31,32,33,34^

### Limitations

This study has limitations. The results of this randomized clinical trial require replication in other care settings and with other groups of patients, especially those who are underserved and uninsured. The study evaluated 1 to 2 primary outcomes in each of the 3 patient cohorts without adjustment for multiple comparisons. The original clinic-randomized study design was disrupted by the COVID-19 pandemic; in the revised study design, each PCC was necessarily unblinded and provided care to patients in both the clinical decision support and usual care groups, with a potential for the intervention to affect care of patients receiving usual care, and thus bias results toward the null.^30,35^ The pharmacist outreach component of the intervention was completed in less than half of patients eligible for outreach.

## Conclusions

In this randomized clinical trial, clinical decision support delivered to PCCs immediately before the clinical encounter, coupled with proactive pharmacist outreach to patients with persistently elevated BP, HbA_1c_, or LDL-C levels and persistent medication nonadherence, had a positive effect on adherence to BP medications. However, statin adherence, diabetes medication adherence, and overall HbA_1c_, BP, or LDL-C control were not improved.

## Notes

Supplement 1.
Study Protocol

Supplement 2. **eMethods.** Detailed Methods

Supplement 3.
Data Sharing Statement
