# Supplementary material for: Clinical Decision Support and Cardiometabolic Medication Adherence: A Randomized Clinical Trial
Source: JAMA Netw Open. 2025 Jan 9;8(1):e2453745. doi: 10.1001/jamanetworkopen.2024.53745 (PMC11718557; doi:10.1001/jamanetworkopen.2024.53745)
Supplement: Supplement 1. — Study Protocol [file jamanetwopen-e2453745-s001.pdf]

Supplementary Online Content

O'Connor PJ, Haapala JL, Dehmer SP, et al. Clinical decision support and cardiometabolic adherence: a randomized clinical trial. *JAMA Netw Open*. 2025;8(1):e2453745. doi:10.1001/jamanetworkopen.2024.53745

eMethods. Detailed Methods

This supplementary material has been provided by the authors to give readers additional information about their work.

© 2025 O'Connor PJ et al. *JAMA Network Open.*

eMethods. Detailed Methods

Electronic Health Record (EHR)-derived Proportion of Days Covered (PDC) measures were computed by comparing each patient's current outpatient medications with the number of times a medication had been dispensed, including the number of days supplied, based on a 6-month observation period prior to the index visit date for each patient to determine study eligibility, using EHR-embedded OPDC algorithms (Epic Systems, Verona, WI). PDC was similarly calculated for each medication of each patient at 6 months, 12 months, and 18 months after the index date, to determine eligibility for pharmacist outreach at 6 months, and to assess medication adherence at 12 and 18 months after index date, which were study outcomes.

Adherence was assessed stepwise as follows: (1) A list of active drugs within each drug class was assembled on the date of interest. Examples of drug classes include statins, biguanide, sulfonylureas, thiazide diuretics, angiotensin converting enzyme inhibitors (ACE) etc. (2) To be classified as "adherent" to each drug class with one or more active drug, at least one of the active drugs within that drug class had to have PDC >= 80%; if so, the patient was classified as "adherent" to that med class at that point in time. (3) Else, the patient was classified as non-adherent to that drug class. (4) If on the date of interest, the patient was on more than one BP (for example) drug class, then the patient was required to be adherent to all BP drug classes active on the date of interest to be classified as adherent to BP meds on that date of interest. (5) The same rules were applied separately for BP meds, glucose-lowering meds, and for statins.

© 2025 O'Connor PJ et al. *JAMA Network Open.*

All the chronic disease care drugs included in the current study are classified as active in the EHR until the earlier of (1) 12 months after the prescription date, or (2) a drug discontinuation date. Patients who received a medication for less than the specified observation window had a PDC calculation based on the length of time the patient was exposed to the medication. Hospital admissions during any lookback period were excluded from the adherence calculation. Dose changes in a medication and the degree of regimen complexity were not accounted for in the PDC calculation. Any duplicate dispense data was removed before the adherence rate was calculated.

Prescription fill data were gathered from multiple sources (administrative claims, pharmacy fills, or recorded samples) and any duplicate dispense data was removed before the adherence rate was calculated. The "fill date" used for the PDC calculation was determined by the data source. Therefore, the fill date was one of the following: (1) the actual pharmacy fill date, (2) the adjudication date, or (3) the prescription pick-up date. Thus, small variations in the PDC calculation were possible.

The EHR-derived PDC measure includes a confidence score calculated for each medication adherence calculation based on the reliability of the source data. For example, medications with dispense history obtained from pharmacy records are regarded with higher confidence than medication dispense records received from recorded samples.

PDC was calculated separately for each statin, each non-insulin glucose-lowering medication, and each BP-lowering medication identified as an active medication at index date or on the date

© 2025 O'Connor PJ et al. *JAMA Network Open.*

6-months, 12-months, or 18-months post-index date. To be classified as adherent to BP, glucose, or statin medications at 12-months or 18-months post-index, all active medications for BP (or glucose, or statins) had to have PDC >=80% with moderate of high confidence to be considered adherent to the relevant category of medication (statins, glucose-lowering medications, or BP-lowering medications). For example, if a patient in the hypertension cohort had an active diuretic and an active ACE prescription, both of those drugs had to have PDC>=80% for the patient to be classified as adherent to BP-lowering medications at the time point of interest. However, if a patient had two active drugs within the same medication class (classes such as statins, sulfonylureas, etc.) then the patient was classified as adherent to that medication class if at least one of the medications within the class had PDC>=80%. For example, if a patient had both glyburide and glimepiride listed as active sulfonylurea medications, and the PDC of glimepiride was >=80% while the PDC of glyburide was <80%, then the patient was classified as being adherent to sulfonylurea medications. This was intended to reduce the likelihood that changes from one medication to another within a class of medications would lead to erroneous classification of the patient as nonadherent to that class of medication.

For determining low adherence, the study used the industry standard PDC threshold of less than 80%. While this PDC cut point is arbitrary, multiple studies have shown that PDC scores less than 80% for cardiometabolic medications may be associated with adverse clinical outcomes.

© 2025 O'Connor PJ et al. *JAMA Network Open.*
