# Supplementary material for: Clinical Decision Support and Cardiometabolic Medication Adherence: A Randomized Clinical Trial
Source: JAMA Netw Open. 2025 Jan 9;8(1):e2453745. doi: 10.1001/jamanetworkopen.2024.53745 (PMC11718557; doi:10.1001/jamanetworkopen.2024.53745)
Supplement: Supplement 2. — eMethods. Detailed Methods [file jamanetwopen-e2453745-s002.pdf]

A Team-Based and Technology-Driven Adherence Intervention to Improve Chronic Disease Outcomes
Short Title: Adherence Wizard

Protocol Number: A16-691

National Clinical Trial (NCT) Identified Number: NCT03748420

Principal Investigator: Dr. JoAnn Sperl-Hillen

Funded by: National Heart, Lung, Blood Institute, National Institutes of Health

Version Number: v.6.0

12th February 2021

Table of Contents

STATEMENT OF COMPLIANCE 1
1 PROTOCOL SUMMARY 1
1.1 Synopsis 1
1.2 Schema 3
1.3 Schedule of Activities (SoA) 4
2 INTRODUCTION 5
2.1 Study Rationale 5
2.2 Background 5
2.3 Risk/Benefit Assessment 10
2.3.1 Known Potential Risks 10
2.3.2 Known Potential Benefits 10
2.3.3 Assessment of Potential Risks and Benefits 10
3 OBJECTIVES AND ENDPOINTS 10
4 STUDY DESIGN 12
4.1 Overall Design 12
4.2 Scientific Rationale for Study Design 13
4.3 Justification for Dose 13
4.4 End of Study Definition 13
5 STUDY POPULATION 13
5.1 Inclusion Criteria 13
5.2 Exclusion Criteria 14
5.3 Lifestyle Considerations 14
5.4 Screen Failures 14
5.5 Strategies for Recruitment and Retention 14
6 STUDY INTERVENTION 15
6.1 Study Intervention(s) Administration 15
6.1.1 Study Intervention Description 15
6.1.2 Dosing and Administration 16
6.2 Preparation/Handling/Storage/Accountability 16
6.2.1 Acquisition and accountability 16
6.2.2 Formulation, Appearance, Packaging, and Labeling 16
6.2.3 Product Storage and Stability 16
6.2.4 Preparation 16
6.3 Measures to Minimize Bias: Randomization 17
6.4 Study Intervention Compliance 17
6.5 Concomitant Therapy 17
6.5.1 Rescue Medicine 18
7 STUDY INTERVENTION DISCONTINUATION AND PARTICIPANT DISCONTINUATION/WITHDRAWAL 18
7.1 Discontinuation of Study Intervention 18
7.2 Participant Discontinuation/Withdrawal from the Study 18
7.3 Lost to Follow-Up 18
8 STUDY ASSESSMENTS AND PROCEDURES 18
8.1 Efficacy Assessments 18
8.2 Safety and Other Assessments 18
8.3 Adverse Events and Serious Adverse Events 18

8.3.1 Definition of Adverse Events (AE) 19
8.3.2 Definition of Serious Adverse Events (SAE) 19
8.3.3 Classification of an Adverse Event 19
8.3.4 Time Period and Frequency for Event Assessment and Follow-Up 20
8.3.5 Adverse Event Reporting 20
8.3.6 Serious Adverse Event Reporting 20
8.3.7 Reporting Events to Participants 20
8.3.8 Events of Special Interest 21
8.3.9 Reporting of Pregnancy 21
8.4 Unanticipated Problems 21
8.4.1 Definition of Unanticipated Problems (UP) 21
8.4.2 Unanticipated Problem Reporting 21
8.4.3 Reporting Unanticipated Problems to Participants 22
9 STATISTICAL CONSIDERATIONS 22
9.1 Statistical Hypotheses 22
9.2 Sample Size Determination 23
9.3 Populations for Analyses 23
9.4 Statistical Analyses 23
9.4.1 General Approach 23
9.4.2 Analysis of the Primary Efficacy Endpoint(s) 24
9.4.3 Analysis of the Secondary Endpoint(s) 25
9.4.4 Safety Analyses 26
9.4.5 Baseline Descriptive Statistics 26
9.4.6 Planned Interim Analyses 26
9.4.7 Sub-Group Analyses 27
9.4.8 Tabulation of Individual participant Data 27
9.4.9 Exploratory Analyses 27
10 SUPPORTING DOCUMENTATION AND OPERATIONAL CONSIDERATIONS 27
10.1 Regulatory, Ethical, and Study Oversight Considerations 27
10.1.1 Informed Consent Process 28
10.1.2 Study Discontinuation and Closure 28
10.1.3 Confidentiality and Privacy 29
10.1.4 Future Use of Stored Specimens and Data 31
10.1.5 Key Roles and Study Governance 31
10.1.6 Safety Oversight 32
10.1.7 Clinical Monitoring 33
10.1.8 Quality Assurance and Quality Control 33
10.1.9 Data Handling and Record Keeping 33
10.1.10 Protocol Deviations 34
10.1.11 Publication and Data Sharing Policy 34
10.1.12 Conflict of Interest Policy 34
10.2 Additional Considerations 35
10.3 Abbreviations 36
11 REFERENCES 38

STATEMENT OF COMPLIANCE

The trial will be carried out in accordance with International Conference on Harmonisation Good Clinical Practice (ICH GCP), United States (US) Code of Federal Regulations (CFR) applicable to clinical studies (45 CFR Part 46, 21 CFR Part 50, 21 CFR Part 56, 21 CFR Part 312, and/or 21 CFR Part 812), and the National Heart, Lung, Blood Institute, National Institutes of Health Research Terms and Conditions of Award.

National Institutes of Health (NIH)-funded investigators and clinical trial site staff who are responsible for the conduct, management, or oversight of NIH-funded clinical trials have completed Human Subjects Protection and ICH GCP Training.

The protocol, informed consent form(s), recruitment materials, and all participant materials will be submitted to the Institutional Review Board (IRB) for review and approval. Approval of both the protocol and the consent form(s) (or waiver of consent) must be obtained before any participant is enrolled. Any amendment to the protocol will require review and approval by the IRB before the changes are implemented to the study. In addition, the IRB will determine whether a new consent is needed based on the approved protocol changes and make a determination regarding whether a recall needs to be obtained from participants who were previously waivered or provided consent.

1 PROTOCOL SUMMARY

1.1 SYNOPSIS

**Title:**	A Team-Based and Technology-Driven Adherence Intervention to Improve Chronic Disease Outcomes
**Study Description:**	More than 50% of adults treated for diabetes, hypertension, or lipid disorders have suboptimal medication adherence, a prominent barrier to continued improvement in chronic disease care in the United States. Primary care providers (PCPs) often fail to identify medication nonadherence and/or have insufficient time and training to address underlying reasons for it. In this project, we propose a patient-centered and technology-driven strategy to identify patients with adherence issues and apply a team approach that will help them achieve evidence-based personalized goals for glucose, blood pressure (BP), or lipids. The study is a two-arm pragmatic patient randomized trial at 28 primary care clinics targeting adult patients not meeting recommended goals for BP, A1C, or statin use with poor medication adherence.
**Objectives:**	(1) Identify and address nonadherence for adults with chronic diseases; (2) Improve management of previously uncontrolled blood pressure, blood sugar, and lipid disorders; and (3) Increase patient involvement in medication decision making.
**Endpoints:**	Primary Endpoint: (a) For each condition, achievement of an Epic generated proportion of days covered (e-PDC) ≥80% for all medication classes for that particular condition (diabetes, hypertension, statin indication) in the 12 months following index date. (b) Mean change in systolic blood pressure (SBP) from the last BP within the 12 months prior to the index visit, to the last BP value within 12 months after the index visit. (c) Change in A1C value from the last lab test within 12 months prior to the index visit to the last lab test within 12 months after the index visit.

	Secondary Endpoints: cost and cost effectiveness of the intervention. Tertiary Endpoints: (a) total 10-year American College of Cardiology/American Heart Association (ACC/AHA) Cardiovascular (CV) risk, (b) patient reported medication adherence using a validated survey instrument, (c) Patient and provider experience and satisfaction, (d) facilitators and barriers to implementation of the intervention
**Study Population:**	The study includes adult patients from at least 28 HealthPartners Medical Group (HPMG) primary care clinics (HealthPartners (HP) and Park Nicollet (PN) in Minnesota.
**Phase:**	3
**Description of Sites/Facilities Enrolling Participants:**	The study will take place at HP and PN primary care clinics who are part of a large integrated care system throughout Minnesota and Wisconsin. Clinics to be enrolled in the study had an affiliation with one of three retail pharmacy chains (HP, PN and Goodrich pharmacies) at the time the study started. A pharmacy affiliation is described as a pharmacy that was located in a HP or PN clinic.
**Description of Study Intervention:**	A clinical decision support (CDS) system for CV risk-factor control will be enhanced to support a team-based care model that does the following: (a) identifies patients not achieving optimal outcomes for BP, lipid, and glucose management, (b) assesses medication adherence information for these patients (c) incorporates adherence information into the clinical decision support tools available at the point of care (d) creates a patient registry that facilitates proactive pharmacist outreach for patients with potential adherence issues identified, and (e) coordinates action plans between pharmacists and other providers on the care team to address adherence issues.
**Study Duration:**	60 months
**Participant Duration:**	12 months minimum follow up after an index visit for a range of 12-18 months follow up.

1.2 SCHEMA

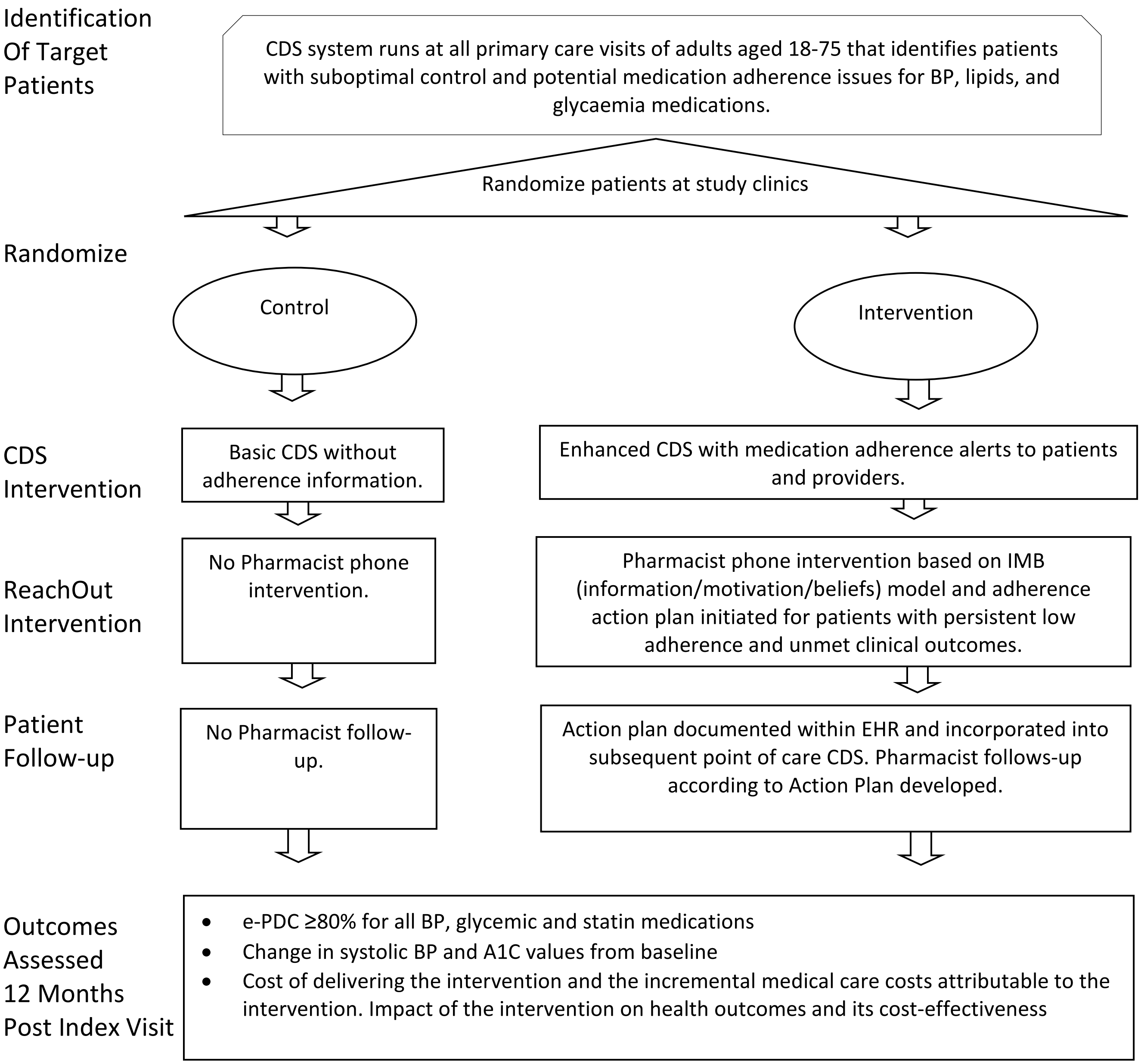
Identification Of Target Patients
CDS system runs at all primary care visits of adults aged 18-75 that identifies patients with suboptimal control and potential medication adherence issues for BP, lipids, and glycaemia medications.
Randomize patients at study clinics
Randomize
Control
Intervention
CDS Intervention
Basic CDS without adherence information.
Enhanced CDS with medication adherence alerts to patients and providers.
ReachOut Intervention
No Pharmacist phone intervention.
Pharmacist phone intervention based on IMB (information/motivation/beliefs) model and adherence action plan initiated for patients with persistent low adherence and unmet clinical outcomes.
Patient Follow-up
No Pharmacist follow-up.
Action plan documented within EHR and incorporated into subsequent point of care CDS. Pharmacist follows-up according to Action Plan developed.
Outcomes Assessed 12 Months Post Index Visit
e-PDC ≥80% for all BP, glycemic and statin medications
Change in systolic BP and A1C values from baseline
Cost of delivering the intervention and the incremental medical care costs attributable to the intervention. Impact of the intervention on health outcomes and its cost-effectiveness

1.3 SCHEDULE OF ACTIVITIES (SOA)

Procedures	Pre-Index Visit	Index Visit – first eligible visit with primary care	0 to 6 months after Index visit	6 to 12 months after index visit	12 to 18 months after index visit
Select study clinics	X				
EHR data is exchanged with Wizard web service in the background at the patient's first and subsequent primary care encounters occurring during the study duration. An encounter-based limited data set is collected and stored in a data repository (intervention & control)	X	X	X	X	X
All eligible patients are randomly assigned a study identification (ID) the first time they are eligible for CDS at a study clinic		X			
Based on the study ID, an eligible patient is then randomly assigned to adherence intervention or control		X			
After Wizard determines study eligibility and CDS recommendations, it prompts CDS tools to be printed for the intervention-assigned patient and their provider through a Best Practice Advisory (BPA) for rooming staff at each primary care encounter during the study period		X	X	X	X
The eligible intervention-assigned patient has an attempted exposure to Adherence CDS at each primary care encounter during the study period		X	X	X	X
Wizard algorithms are run outside of encounters on all study eligible patients at set intervals (6, 12, and 18 months) for analysis purposes. A limited data set is collected and stored in a data repository (intervention & control patients)			X	X	X
An eligible intervention-assigned patient is added to a pharmacist outreach intervention registry if study criteria are still met (not at goal and not adherent)			X		
An eligible intervention-assigned patient is assigned to a pharmacist			X		
Pharmacist calls eligible patient (multiple attempts) for adherence assessment and action plan				X	
Pharmacist follow-up per action plan				X	X
Experience and satisfaction surveys sent to a random group of patients (intervention & control)			X	X	

Procedures	Pre-Index Visit	Index Visit – first eligible visit with primary care	0 to 6 months after Index visit	6 to 12 months after index visit	12 to 18 months after index visit
Safety monitoring per Data Safety Monitoring Board (DSMB) plan (intervention & control)	X	X	X	X	X

2 INTRODUCTION

2.1 STUDY RATIONALE

More than 50% of adults treated for diabetes, hypertension, or lipid disorders have suboptimal medication adherence, a prominent barrier to continued improvement in chronic disease care in the United States (US). Primary care providers (PCPs) often fail to identify medication nonadherence and/or have insufficient time and training to address underlying reasons for it. In this project, we propose a patient-centered and technology-driven strategy to identify patients with adherence issues and apply a team approach to help them achieve evidence-based personalized goals for glucose, blood pressure, or lipids. This intervention extends the use of a widely available clinical decision support (CDS) infrastructure to support a model of care that will integrate pharmacists within the primary care team. The intervention relies on a continuous health informatics loop to do the following: (a) identify poor adherence to medications; (b) establish and maintain a registry of patients identified for proactive pharmacist outreach; (c) implement a pharmacist outreach strategy based on an information/motivation/behavioral (IMB) framework recommended by the World Health Organization (WHO) with demonstrated ability to influence adherence across a variety of clinical applications; and (d) coordinate care and adherence information by incorporating pharmacist assessment and action plans into CDS at subsequent office encounters.

2.2 BACKGROUND

<u>The Consequences of Medication Nonadherence</u>

Medication adherence has been shown to be suboptimal across multiple chronic disease states[1-4] and is a key factor that affects clinical outcomes.[2,5-11] It is estimated that 50% of chronic disease medications are not taken as prescribed,[12] leading to compromised health, increased hospitalizations and mortality, and serious economic consequences.[3,9,13-22] Medication non-adherence costs the US $258 to $290 billion annually, thus contributing substantially to the current health care cost crisis[23-25]. The clinical consequences of medication non-adherence include an estimated 125,000 deaths a year, and contributes to millions of potentially preventable hospital admissions each year.[3,26,27]

Determinants of poor medication adherence are complex and include health system, social/economic, therapy-related, condition-related, and patient-related factors.[28,29] Because nonadherence involves a complex cluster of behaviors, many different constructs have been developed to identify patients and quantify problems related to patients who have been prescribed a drug and have not filled a prescription or are not using the drug as prescribed, whether intentionally or non-intentionally.[30-35]

<u>Challenges of Measuring Nonadherence</u>

In outpatient settings, patient self-reported medication adherence measurements can be time consuming to obtain, are subject to recall bias and overestimation of adherence, and have a propensity

to elicit socially acceptable responses that may not correlate well with refill and administrative measures.[36,37]Objective measures of adherence derived from administrative claims and electronic medical record (EMR) data are more efficient and less biased. A *variety of such measures have been developed*,[38] and standardized terminology and definitions have recently been advocated.[39] The Proportion of Days Covered (PDC) has been operationalized and endorsed by the Pharmacy Quality Alliance and introduced by the Centers for Medicare & Medicaid Services (CMS) as a quality metrics tool to assess secondary adherence related to hypoglycemic, antihypertensive, and cholesterol-lowering drugs. The PDC reduces overestimation of adherence, eliminates potential recall bias associated with patient self-report, and provides a conservative estimate of the highest possible level of medication consumption.[38,40] For this study, limitations of PDC include (a) the data is only available for patients with claims data through HealthPartners insurance, and (b) PDC is not useful for calculating primary nonadherence (a medication prescribed but never filled) because the prescribed medication will not be identified in claims data.[39]

In the 2018 version of Epic (Epic Systems Corporation), a measure of adherence similar to a calculated PDC is made available at encounters based on the total number of days supplied for a specified measurement period of time (default time being 6 months) prior to a clinical encounter. According to Epic, 'Medication adherence is determined by comparing a patient's current outpatient medication list in Epic with the number of times a medication has been dispensed.'[41] In Epic, PDC is expressed as the percent of days in the measurement period before the encounter covered by medication fills for a specific medication based on data from Surescripts, an information technology company that supports e-prescription with the most comprehensive prescription dispense data in the United Sates. An indicator of confidence in the e-PDC (high, medium, or low) is also determined and displayed with the e-PDC score based on a determination of how accurate or reliable the Surescripts data is. The confidence is high if a patient fills all their prescribed medications from a pharmacy that reports all their dispense data to Surescripts; medium if high criteria is not met but there is some dispense and claims data available; low if a patient fills their prescribed medications from different pharmacies, some of which do not report to Surescripts and no claims data is available[41].

For this intervention, the Epic pre-calculated PDC score has the advantage of being available for most patients seen in the care system rather than being limited to a subset of patients with HealthPartners insurance claims data available, which would be the case if we were to calculate PDC ourselves in the traditional methods using claims data. Because of the clinical advantages of e-PDC over calculated PDC, the MAS has been chosen for this study as the objective measure of medication adherence for intervention and study outcomes.

<u>The New Care Paradigm includes Pharmacists</u>

When medications are prescribed and monitored, physicians often fail to communicate and discuss with patients critical elements about medication use. Thus, the adherence problem will be hard to solve by expecting more adherence and medication counseling to be done by physicians who often lack the time and may lack appropriate training to provide adherence counseling[42-45]. Previous research shows that community-based pharmacists are well-trained and well-positioned to provide medication support for people with chronic conditions.[46,47] A 2011 report to the US Surgeon General urged moving beyond research to widespread implementation and adoption of a care model that embraces embedded pharmacist services.[48] Since 2004, pharmacist licensure requires a Doctor of Pharmacy degree, which is a four-year academic program designed to ensure competency of pharmacists as medication managers. Pharmacists coming out of training are eager to provide these enhanced services with 95% of student pharmacists believing that providing clinical services is important and 87% believing that clinical care

opportunities exist for them in the workplace.[49] Opportunities for clinical care involvement are growing nationally with many rigorous demonstrations of the clinical and economic value of pharmacists. [49-55] HealthPartners has also embraced the expanded role of pharmacists and we have shown in a randomized trial that home BP telemetry with pharmacist case management significantly improved BP control in hypertensive adults compared with usual care after 6 and 12 months.[50] A recent meta-analyses supports the ability of pharmacists, alone and as part of a team-based care model, to improve medication adherence and control of hypertension[51] and CV risk factors in patients with and without diabetes. There are numerous other examples of coordinated care models in the US that have removed barriers to pharmacist participation: (1)The Asheville Project model which began in 1997 incorporated fee for service payments from employers to pharmacists and saved North Carolina $13 million.[52,53] The model was expanded within the US through the 10-City Diabetes Challenge Project in 2007.[54] (2) The Wisconsin TEAM Trial demonstrated the ability of pharmacists to improve medication adherence and BP control in patients with hypertension.[55] (3) The Pennsylvania Project demonstrated that community pharmacists improved PDC for 5 targeted medication classes and reduced total health care costs ($241 for patients taking statins; $341 for patients taking diabetes medications).[56]

The Joint Pharmacist Organization Committee representing over 100,000 pharmacists across a spectrum of practice settings is actively involved in policy decisions to ensure that pharmacist payment structures keep pace with the evolving value-based health care system and pharmacy profession.[57] Patient-centered medical homes (PCMH) within the Veterans Affairs (VA) Health Care System and Maine PCMH are examples of how pharmacists can be used within value-based payment models.[58] The 2016 CMS Transforming Clinical Practice Initiative creates US networks to promote the establishment of community based health teams to support chronic care management.[59]

Pharmacists are perhaps the most accessible of all health professionals and are routinely ranked as one of the most trusted professionals in the United States.[60,61] Further, patients are more willing to follow through with prescriber recommendations when reinforced by pharmacistss.[62] With growing pharmacist access to the EMR, integration of pharmacists into the care workflow, and stronger communication with prescribers, there is reason to expect a substantive favorable effect of increased pharmacist involvement on medication adherence and on chronic disease outcomes.[63-65]

Clinical Decision Support Systems

EMR-based CDS systems initially were limited to prompts and reminders, which had a favorable impact on ordering of tests and procedures but failed to improve intermediate outcomes of care such as BP, glucose, or lipid control.[66] However, in the last decade, EMR-based CDS systems have become much more sophisticated and have been shown to improve BP and glucose control and other intermediate outcomes of care in numerous studies.[67-70] However, to date, only a few integrated health systems that include both claims and clinical data (e.g., Kaiser) have been able to incorporate medication adherence data into the EMR and use those data in a sustained way.[71] Furthermore, incorporation of adherence-related data into EMR dashboards and CDS tools thus far has not been shown to have a significant impact on clinical outcomes.[72] This is not surprising, because prescribers often lack the time and training to skillfully address medication adherence issues in the context of a visit. In fact, some research suggests that prescribers supply vital information about new prescriptions less than 30% of the time.[44,45] Thus, most studies of CDS that include adherence data have been limited in impact by underutilization of the tools and/or unsuccessful reliance on patients to complete adherence barriers assessments through patient portals.[72] One obvious improvement opportunity is to integrate pharmacists into the care process. However, although it is operationally feasible, most community pharmacists currently do not have access to the EMR so that (a) two-way communication between PCPs and pharmacists is

inefficient, (b) the prescriber has limited fill data to use for assessing adherence, or is unaware of the availability and interpretation of available e-PDC and (c) it is difficult to integrate pharmacists working remotely into primary care teams. This project squarely addresses the barriers to integration of pharmacists into primary care teams outside of totally integrated care systems (about 90% of Americans). The challenges are solvable using novel informatics approaches. In this project, we use an established multifunctional CDS system as the cornerstone of an expanded informatics system that (a) makes adherence data available to prescribers at the time of a visit, (b) enables efficient and automatic bidirectional communication between pharmacist and prescribers, and (c) directs pharmacist attention to the subset of non-adherent, poorly controlled patients who are most likely to benefit from personalized attention to adherence-related issues.

<u>The Information-Motivation-Behavior (IMB) conceptual model for the pharmacists' intervention</u>

The IMB conceptual model that guides this intervention (Figure below) posits that information is a prerequisite for changing behavior but by itself is insufficient to achieve behavior change.[73] Motivation is also a key determinant for behavior change and has been found to be a key factor in promoting adherence to chronic therapies, but the relationship between motivation and information constructs is elastic (a highly informed person may have low motivation and vice versa). Practically, the IMB model directs the pharmacist's attention to the influence of psychosocial factors related to adherence behaviors, including readiness to change, that evolve over time and cannot be addressed by information interventions alone. Interventions based on IMB have been effective at influencing behavioral change across a variety of clinical applications, including adherence to asthma medication and reduction of HIV risk behaviors.[74-78] The IMB model incorporates elements of social cognitive theory (the importance of self-efficacy), stress coping model (dealing with the stress of the condition), and trans-theoretical model (incorporating readiness to change) – all of which are evidence-based and considered important for successful self-management interventions for people chronic disease.[79-83] The IMB model has been endorsed by the WHO to address adherence problems, with recognition that the presence of both motivation and information increases the likelihood of adherence.[28,84]

Figure 1: Information-Motivation-Behavior Change Framework

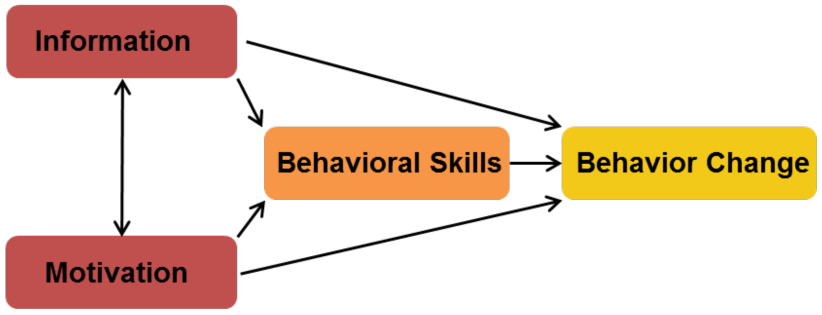

Source: World Health Organization, 2003

<u>Experience with CDS Development and Implementation</u>

We previously constructed and evaluated an EMR-based diabetes CDS system to improve outcomes in adults with type 2 diabetes in an NIH-funded clinical trial (DK068314). In a randomized trial of 11 clinics with 41 consenting PCPs and 2556 eligible diabetes patients we showed significantly better A1C (intervention effect -0.26%, 95% CI: -0.06 to -0.47, P=.014) and systolic BP (intervention effect -2.64 mm Hg, 95% CI: - 0.19 to -5.10, P=.035) in intervention group than control group patients using a general linear mixed-model analysis with repeated time measurements and control for clustering. Low-density

lipoprotein (LDL) cholesterol improved a mean of 23 mg/dL in both groups but was not superior in the intervention group (*P*=.63).[84,85] Over 94% of PCPs expressed satisfaction with the CDS.[86] In subsequent National Heart, Lung, and Blood Institute (NHLBI) funding (R01HL102144), we expanded the CDS tool to focus on CV risk factor domains, and we improved the workflow and scalability in significant ways.

The current version of this CDS system is Web-based for more efficient scalability and dissemination to other EMR systems, and we prioritize care recommendations based on potential CV benefits and severity of clinical conditions. Moreover, CDS is provided both to the provider and directly to the patient in order to promote patient engagement and shared decision making. The patient-directed CDS display presents sophisticated risk information in a low literacy and numeracy format.[87-90] PCPs use this CDS at more than 75% of targeted office visits. The CDS system has now been disseminated to all clinics in HPMG (HP and PN) and to 2 large rural care systems and is used on a daily basis in the care of more than 1.5 million patients in four states. Our extensive experience and prior work enables us to immediately focus on the substantial enhancements needed to integrate medication adherence data and pharmacist outreach components into the underlying CDS system, and to assess impact on care.

<u>Experience with pharmacists on the care teams</u>
Pharmacists have already begun to embrace system wide care strategies to expand the pharmacist role to support the care of patients with targeted conditions. Some pharmacists have bidirectional access to the care system's EMR in compliance with patient privacy regulations and have already been trained to conduct targeted EMR chart reviews, order overdue labs based on standing orders and care protocols, and reinforce diabetes clinical goals when processing prescriptions for diabetes medications or supplies. Additionally, some pharmacists use protocol-driven standing orders to adjust hypertension therapy and authorize refills for chronic conditions. These pharmacists are enthusiastic about doing clinical work, and seek out roles that allow them to focus on patient care. The care system is engaged and supportive of this research project and anticipates a more active future role of pharmacists on the care team.

<u>Preliminary Pilot Data</u>**:** Supporting data for this were obtained from a repository of CDS-generated, EMR-based data that included HealthPartners and Park Nicollet affiliated clinics. We evaluated 26,351 encounters among 19,976 patients aged 18-75 from 28 clinics with affiliated pharmacies for the period January 16, 2019 to April 25, 2019. The data included a comprehensive list of medications (with medication identifiers) for BP, lipids, and glycemic control and algorithmically derived information related to clinical conditions, lab measures, and e-PDC.

Table 1. Primary Care Patients with Chronic Conditions with Adherence Issues Identified, 1/16/19 – 4/25/19

Number of patients with the condition identified at a visit	On medication related to the condition	Meeting clinical eligibility criteria[a]	e-PDC confidence moderate/high	e-PDC 1-79%	Primary care visit
Hypertension N=13,349	N=12,360 (93%)	N=2,891 (23%)	N=1,808 (63%)	N=816 (45%)	N=684 (84%)
Diabetes N=12,158	N=8,827 (73%)	N=3,385 (38%)	N=2,240 (66%)	N=1,123 (50%)	N=983 (88%)
On statin N=11,085	N=11,085 (100%)	N=10,645 (96%)	N=6,565 (62%)	N=2,334 (36%)	N=2,010 (86%)

[(a)] A1C $\geq$8%, BP $\geq$140/90 mm Hg on 2 consecutive occasions or meeting ACC/AHA indications for moderate or high intensity statins.

Table 1 indicates the total number of patients identified with hypertension (n=13,349), diabetes (n=12,158), or statin use (n=11,085). Over 11,000 of these patients are not meeting recommended clinical goals and 21% to 33% of these have secondary nonadherence issues identified.

2.3 RISK/BENEFIT ASSESSMENT

2.3.1 KNOWN POTENTIAL RISKS

Potential risks to patient participants include the possibility that the intervention may provide treatment considerations to PCPs on the basis of the national evidence-based guidelines and/or national standards for adherence measurement that may be inappropriate for a given individual patient and, if applied without further checking the clinical status of a given patient, could lead to erroneous therapy, adverse events, disability, or death. However, the clinical recommendations are evidence-based and operationalize current national and regional standards of care; therefore, the risk of untoward consequences of such clinical actions is considered minimal. Moreover, this potential risk is routinely present in every clinical encounter in the health care system.

2.3.2 KNOWN POTENTIAL BENEFITS

Patient participants will have no defined personal benefit from participating in this project. Other than surveys and maybe interviews in a sample of study eligible patients, no direct communication between research team members and patient participants is planned as part of the study protocol. Although some patients with adherence issue and sub-optimally controlled cardiovascular risk factors and diabetes may be identified better by their care providers as a result of this intervention, no claim of clinical benefit to an individual patient can or will be made.

2.3.3 ASSESSMENT OF POTENTIAL RISKS AND BENEFITS

The implementation of clinical decision support and pharmacist outreach related to medication adherence for chronic disease management poses minimal risk to providers and patients. The CDS recommendations are concordant with relevant clinical practice guidelines and facilitates provider access to clinically relevant information at the point-of-care. The CDS intervention training and statements on the CDS displayed will reinforce that providers should exercise their judgement before following any CDS treatment suggestions. Therefore, patients will be exposed to standard of care. One risk in this study is the potential breach of privacy, for which appropriate measures have been taken to mitigate the possibility of such risk. The potential for these interventions to improve clinical outcomes and increase medication adherence for patients with suboptimally controlled chronic conditions has important public health implications that outweigh the potential risks.

3 OBJECTIVES AND ENDPOINTS

OBJECTIVES<br>What are we measuring	ENDPOINTS<br>How are we measuring it?	JUSTIFICATION FOR ENDPOINTS
Primary		
1. Impact of the intervention on **blood pressure** medication adherence and	1. We will assess patient achievement of (a) an **e-PDC** ≥80% for all BP medications and (b) a reduction in **systolic BP**. (Time Frame is 12-month period after an index office visit.)	The e-PDC has been operationalized by the Epic (Epic Systems Corporation) and made readily available in their 2018 EMR version. This e-PDC is available for

OBJECTIVES<br>What are we measuring	ENDPOINTS<br>How are we measuring it?	JUSTIFICATION FOR ENDPOINTS
systolic blood pressure control<br><br>2. Impact of the intervention on non-insulin **glycemic** medication adherence and A1C control<br><br>3. Impact of the intervention on **statin** medication adherence	**2.** We will assess patient achievement of (a) **e-PDC**≥80% for all non-insulin glycemic medications and (b) a reduction in **A1C** values. Time Frame is 12-month period after an index office visit.<br><br>**3.** We will assess patient achievement of an **e-PDC** ≥80% for a statin medication. (Time Frame is 12-month period after an index office visit)	prescription medication and provides current levels of adherence as well as a measure of the quality of data used to calculate the score expressed as the confidence level. The e-PDC reduces overestimation of adherence, eliminates potential recall bias associated with patient self-report, and provides a conservative estimate of the highest possible level of medication consumption per the available data at the moment.<br><br>**Systolic blood pressure, A1C, and statin use** are intermediate variables strongly associated with morbidity and mortality related to the associated clinical domains.
Secondary		
1. Impact of the intervention on **overall healthcare costs**<br><br>2. Predicted **long-term health impact and cost-effectiveness** of the intervention from the health system (payer) perspective	1. We will measure the cost of delivering the intervention using micro-cost accounting methods and the incremental medical care costs attributable to the intervention, defined from the health system perspective, measured using insurance claims incurred in the median 12-month pre- and post-index date periods by participants with an affiliated HealthPartners insurance plan in each study group.<br><br>2. We will use a cardiovascular disease microsimulation model to predict the impact of the intervention on health outcomes and its cost-effectiveness, defined from the health system perspective, over up to a 30-year prospective time horizon.	1. If the intervention is found to be effective, the cost of delivering the intervention and its incremental effect on medical care utilization will be important outcomes to informing dissemination and implementation to other systems and settings.<br><br>2. A full measurement of the value of the intervention cannot be observed during the study period, but validated microsimulation methods can be used to estimate the full impact of the intervention on preventing or delaying incidence of disease and in averting future medical care costs—both of which will be important to inform the translation and adoption of the intervention, if found to be effective.
Tertiary/Exploratory		

OBJECTIVES<br>What are we measuring	ENDPOINTS<br>How are we measuring it?	JUSTIFICATION FOR ENDPOINTS
1. The impact of the intervention on 10-year CV risk<br>2. Survey assessed patient reported adherence<br>*3.* Patient and provider experience and satisfaction<br>*4.* Qualitative analysis of CDS user experience	1. We will assess reduction in 10-year CV risk measured at each encounter<br>2. We will conduct phone surveys on a random sample of up to 1400 eligible patients approximately 2-4 weeks after an index visit and subsequently ~6 months later (after a phone outreach has occurred)<br>3. We will survey all providers and pharmacists approximately 6-12 months after implementation<br>4. We may conduct end user interviews	The CDS assesses and prioritizes CV risks factors according to the percentage of 10-year CV risk that can be reduced if actions are taken to address the risk factor.<br><br>Provider and patient experience and satisfaction information provide end-user opinions of the intervention which helps to assess impact and improve the product.

4 STUDY DESIGN

4.1 OVERALL DESIGN

- *A statement of the hypothesis:* Poor adherence to medications for chronic diseases is common, but providers often fail to identify patients with adherence issues and/or lack adequate time to address them. We hypothesize that the intervention will proactively (1) identify and address nonadherence for adults with chronic diseases; (2) improve management of previously uncontrolled blood pressure, blood sugar, and lipid disorders; (3) increase patient involvement in medication decision making; and (4) develop informatics systems that integrate retail pharmacists within the primary care team.
- *Phase of the trial:* 3
- *A description of the type/design of trial to be conducted:* This study is a prospective, 2-arm, trial randomizing patients receiving care at primary care clinics to Adherence intervention (ReachOut) or control.
- *A description of methods to be used to minimize bias:* Patient randomization using a computer generated allocation is used to minimize selection bias.
- *The number of study groups/arms and study intervention duration:* The study has 2 arms, intervention and control. The minimum intervention duration is 18 months (about 6 months of accrual of patient index visits and a minimum of 12 months follow up after the index date).
- *Indicate if single site or multi-site:* Single site
- *Name of study intervention(s):* ReachOut Adherence Intervention (clinical decision support plus pharmacist outreach)
- *Name of sub-studies, if any, included in this protocol:* None

4.2 SCIENTIFIC RATIONALE FOR STUDY DESIGN

This trial was originally conceived and approved as a clinic cluster randomized trial of control (usual care) versus intervention. However, the intervention was suspended in March 2020 after one week of implementation due to COVID-19 pandemic response that resulted in loss of the separation of treatment groups. There were clinic closures, decreased office visits, and increase in telehealth care. To address concerns related to patient crossover from intervention to control settings (and vice versa) in the ongoing COVID-19 era, the study was transitioned to a patient level randomization that was conducted using a computerized allocation by the CDS at the time of an index visit within any of the 28 study clinics. The main advantage to the patient level randomization is that no matter what happens with clinics closing and opening, provider moving between clinics, and where video encounters take place, the study subjects consistently receive the ReachOut intervention or usual care without crossover. Additionally study power increases with the patient-level design compared to a clinic-randomized design. The disadvantage to the patient level randomization design, and why it wasn't the initial choice when the grant was originally funded, is concern over clinician contamination due to a potential learning effect from the intervention that translates to control patients. However, the net effect of this contamination would be a reduction in the intervention effect which would not undermine conclusions if the intervention is found to be effective. Given how the clinical setting changed abruptly and unexpectedly due to the COVID-19 pandemic, the advantages of a patient-level study design were determined to outweigh the disadvantages and be the most appropriate way to complete the study and test the hypotheses.

4.3 JUSTIFICATION FOR DOSE

Not applicable

4.4 END OF STUDY DEFINITION

The end date for the study is defined as 12 months after the last patient index visit during the 6 month accrual period.

5 STUDY POPULATION

5.1 INCLUSION CRITERIA

To be eligible for the study, the patient must have an office visit with a clinician (physician or advanced practice provider) within a primary care department (family medicine, internal medicine, combined pediatric/internal medicine) at one of the 28 study clinics within the HealthPartners Medical Group or Park Nicollet care system, and meet all of the following criteria:

1. Age 18 to 75 years, inclusive. The evidence-based guidelines on which the CDS intervention is based are not applicable outside this age range.[91]

2. Have a primary care encounter (index visit) at a selected clinic.

3. Have a primary care encounter that meets one of or more of the following clinical criteria for care goals and medication adherence:

   1. In the 12 months prior to the index visit, most recent A1C ≥8% AND have one or more active non-insulin glycemic medications on their EMR medication list AND a potential adherence issue for one or more of these medications based on the Epic Medication Adherence score (e-PDC <80%, moderate or high confidence)

2. Two consecutive encounters with BP values ≥140/90 mm Hg AND one or more BP medications on their EMR medication list AND a potential adherence issue identified (e-PDC <80%, moderate/high confidence)

3. Meet the ACC/AHA criteria listed below for moderate or high-intensity statin use AND a statin medication on their EMR medication list AND a potential statin adherence issue identified (e-PDC <80%, moderate/high confidence):
    a. Age >21 with atherosclerotic CV disease identified by a CVD diagnosis on the problem list or two or more ICD-10 diagnostic codes in the last 2 years
    b. Aged >21 and LDL >190 mg/dl
    c. Aged 40 to 75 AND diabetes identified by the diagnosis on the problem list or two or more ICD-10 diagnostic codes in the last 2 years
    d. Aged 40 to 75 with 10-year CV Risk Score ≥7.5% based on the ACC/AHA 10-year ASCVD risk equation.

5.2 EXCLUSION CRITERIA

An individual who meets any of the following criteria will be excluded from the study analysis:

1. Patients enrolled in hospice,
2. Patients with active cancer or undergoing chemotherapy
3. Patients with pregnancy identified in the last year
4. Patients without HealthPartners insurance coverage for at least 30 continuous days in the 12 months before or 12 months after the index visit will be excluded from the cost analysis.
5. For Statin cohort, most recent LDL result <100 mg/dl in the last 2 years

5.3 LIFESTYLE CONSIDERATIONS

Not applicable

5.4 SCREEN FAILURES

Not applicable. There are no screening procedures for this pragmatic trial. All patients meeting inclusion and exclusion criteria in selected study clinics are eligible for the intervention.

5.5 STRATEGIES FOR RECRUITMENT AND RETENTION

The study is endorsed by medical leadership and study clinics included had leadership approval. The CDS system has become part of the routine care and workflow for patients in the clinics and CDS content is based on pre-approved evidence based guidelines. As such, consent is waivered for providers and patients.

Several strategies used successfully in previous studies will be adopted to ensure high use rates of the CDS at the point of care. These include:
1. Training sessions for clinic staff by webinars (with lunch provided) prior to the onset of the intervention to stress the importance to patients and recommended workflow. Communication to clinic staff and clinic leadership through care system newsletters and direct emails to stress the importance of the intervention to patients and the usefulness to providers.
2. Reliance of nursing staff rather than providers to print the CDS tools when a BPA pops up during the rooming process for patients who meet the study edibility criteria.

3. Sending clinic leaders print rate reports at the care system, clinic, and provider levels for rates of printing the CDS tools for eligible patients, with a goal of print rates >75%.
4. Troubles shooting phone calls and emails to clinic leaders if use rates drop below goal.

For the pharmacist outreach part of the intervention, we will use a registry system to track outreach phone calls from start to completion. We will work with pharmacy leadership to develop strategies to improve patient contact rates as needed.

6 STUDY INTERVENTION

6.1 STUDY INTERVENTION(S) ADMINISTRATION

6.1.1 STUDY INTERVENTION DESCRIPTION

Control: All control patients will continue to receive the basic EMR-linked CDS for management of CV risk factors and other chronic conditions. This CDS has been described in detail in previous publications, and includes algorithmically derived identification of high CV risk patients and prioritized treatment suggestions for lipids, BP, glycemic control, weight, tobacco, and aspirin use based on distance from goal, current medications, labs, allergies, and safety considerations.[85,92-97] The basic CDS does not include information on medication adherence.

Intervention: For intervention patients, the basic CDS system for CV risk-factor control is enhanced to support a team-based care model that identifies risk of nonadherence, incorporates medication adherence information into the CDS, creates a registry to direct proactive pharmacist outreach, and coordinates action plans (Figure below).

Figure 2: Operational Model for Adherence Intervention

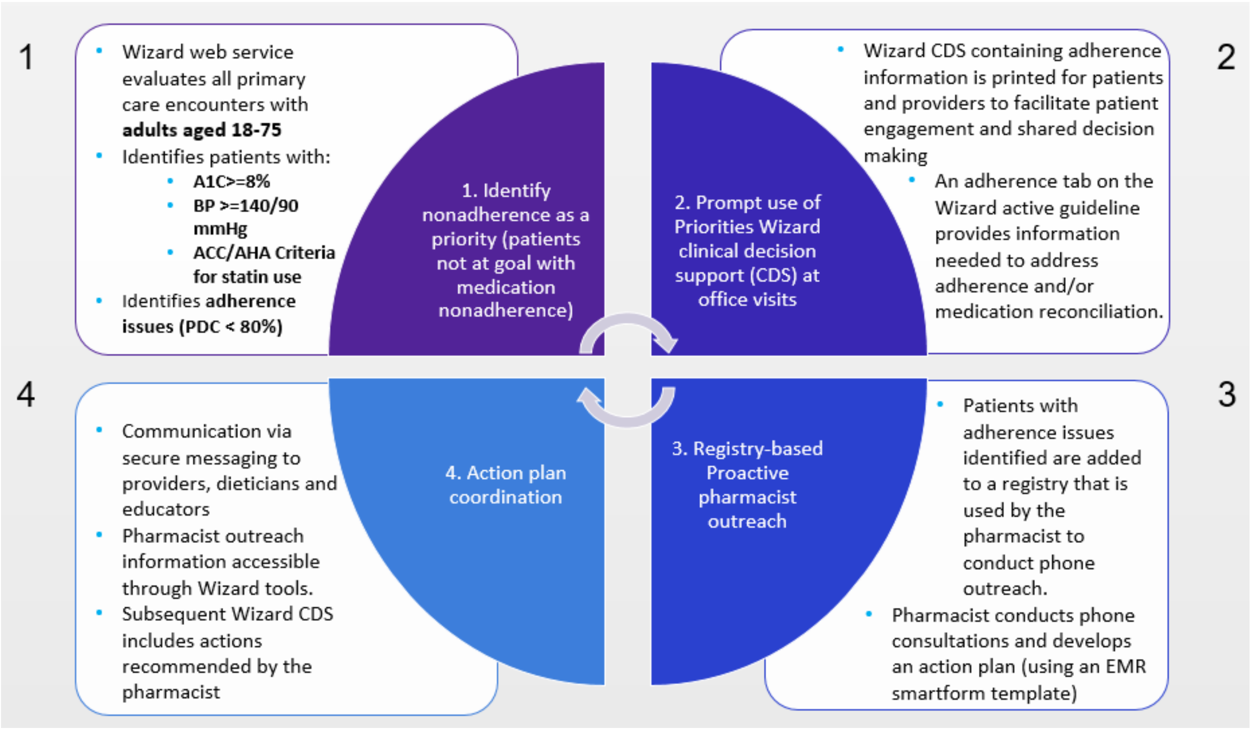

To do this, the CDS Web service will combine EMR-identified medications with e-PDC data. Algorithms will identify poor adherence using e-PDC scores available within the Epic EMR at patient encounters. When medication adherence issues are identified, alert messages will appear on the CDS tools for patients and providers. Patients with index visits will be followed-up for 6 months as they continue to receive CDS at subsequent encounters. At 6 months, patient e-PDC as well as clinical outcomes will be reevaluated.

Patients with persistent adherence issues identified are added to a registry that is used by the pharmacists to conduct outreach. Pharmacist outreach will be conducted primarily by phone, but in-person arrangements are also an option. Pharmacists conducting outreach will identify themselves to patients as part of the care team working with the PCP. The IMB model described in detail in Section 2.2 will be the framework for assessing and addressing adherence. Examples of specific action plans that may be recommended includes education, recommending lower cost alternative medications or combination medications, addressing side effects, using pill boxes, modifying pill-taking schedules and/or using reminder systems, or referrals to MTM pharmacist services or health educators. Pharmacists will be guided by a script template for the phone outreach that walks them through the IMB intervention and data collection. The pharmacists involved will have full read/write access to the EMR in full compliance with HIPAA regulations. They can make medication changes through established care protocols and/or communicate with the prescriber through secure messaging or phone consultation. The date of the pharmacist outreach and actions that result from the IMB intervention will be documented in the EHR and incorporated into subsequent CDS tools and registries to reflect the new patient state.

From preliminary data collected at these clinics, we expect that subjects meeting eligibility requirements will average 3.5 visits per year to their PCP. Moreover, others and we have shown that adherence varies within patient over time[92,98-103], and this intervention provides an efficient and powerful tool for ongoing surveillance of adherence for timely identification of lapses in adherence, with tailored CDS based on systematic assessment of IMB factors related to relapses in adherence.

6.1.2 DOSING AND ADMINISTRATION

Not applicable

6.2 PREPARATION/HANDLING/STORAGE/ACCOUNTABILITY

6.2.1 ACQUISITION AND ACCOUNTABILITY

Not applicable

6.2.2 FORMULATION, APPEARANCE, PACKAGING, AND LABELING

Not applicable

6.2.3 PRODUCT STORAGE AND STABILITY

Not applicable

6.2.4 PREPARATION

Not applicable

6.3 MEASURES TO MINIMIZE BIAS: RANDOMIZATION

Patients are randomly allocated 1:1 through a computer-generated program to either control or intervention. Contamination across intervention arms is unlikely because all patients receive CDS interventions based on their arm assignment. All patients assigned to the study arms are analyzed based on their arm assignment.

6.4 STUDY INTERVENTION COMPLIANCE

Training

Intervention pharmacists will be compensated to attend a 2-3-hour training session to learn the IMB framework, modules related to the chronic diseases addressed, interviewing techniques, and how to use the registry and EMR tools for tracking outreach attempts and documentation. All training will be conducted collaboratively with Goodrich, HealthPartners and Park Nicollet pharmacy directors and managers and the research team. The training format will be modeled after successful pharmacist training programs previously conducted in our care system by pharmacy leadership for diabetes. Training materials will include written materials with companion online and Web-based modules related to the diseases addressed and the IMB intervention. Targeted individual follow-up training may be provided as needed. For PCPs and clinic staff, we will conduct luncheon training sessions (providers, nurses, pharmacists, and rooming staff) either in-person or through webinars that focus on workflow and enhancements to the basic CDS system to address medication adherence. We will also send information through newsletters and communication via email to clinic leaders through the care system leadership.

Follow-up

Rates of PCP use of the CDS and rates of completed pharmacist outreach will be monitored continuously during the study period (through electronic reports) and reported monthly to clinic and pharmacy leadership. If needed to increase use rates and consistent with our previous successful CDS implementation, small incentives (e.g. $250 per clinic semi-annually) may be provided to clinics whose PCPs achieve CDS use rates at >75% of eligible encounters in both intervention and control sites.

The outreach, including the percent of patients with completed outreach contact will also be tracked. Per agreement with the participating pharmacies, we will provide compensation for time spent implementing the outreach. If necessary, trouble shooting with pharmacy leaders and/or supplemental training will be provided to pharmacists with low completed contact rates. While the proposed intervention is rooted in the pragmatism of the current health care world, it is also based on an established process-control conceptual model, current theories of behavior change, and our previous work on chronic disease prevention and control in the study population.[104-106]

Study Team Monitoring

Co-Investigators will regularly meet with study staff to discuss study progress and address potential issues. The purpose of these meetings is to focus on the day-to-day operations of the project and to assure that all necessary tasks are completed in a timely fashion and strictly according to study protocol. Quarterly meetings will include steering group members and will address scientific issues, including refinement of conceptual models, strategies to streamline and deploy the interventions efficiently and effectively, and strategies to maximize both recruitment and retention of study subjects, as well as methods to assure uniformity and fidelity to intervention protocols and data collection.

6.5 CONCOMITANT THERAPY

Not applicable

6.5.1 RESCUE MEDICINE

Not applicable

7 STUDY INTERVENTION DISCONTINUATION AND PARTICIPANT DISCONTINUATION/WITHDRAWAL

7.1 DISCONTINUATION OF STUDY INTERVENTION

This is a minimal risk study without need for halting rules. The CDS suggestions are evidence-based and guideline driven and providers are always advised to exercise their clinical judgement with any CDS suggestions.

7.2 PARTICIPANT DISCONTINUATION/WITHDRAWAL FROM THE STUDY

Providers and patients exposed to the clinical decision support can ignore or disregard any component of the intervention at their discretion. Patients can decline or refuse phone calls from pharmacists. Patients who opt-out of participating in research will be excluded from the analysis dataset.

7.3 LOST TO FOLLOW-UP

All eligible patients who are enrolled in the analysis at an index visit will be included in the analysis (unless they have opted out of research). Patients who move, disenroll from the clinic, or have no follow up in the clinic will be accounted for through the analytic plan. We anticipate loss to death < 2% and disenrollment from the insurance plan in < 5% per year. Patients who have opted out of research per federal law are excluded from the analysis (<2%). Few patients transfer care between HealthPartners and/or Park Nicollet clinics.

8 STUDY ASSESSMENTS AND PROCEDURES

8.1 EFFICACY ASSESSMENTS

Rates of use of the CDS and rates of completed pharmacist outreach will be monitored continuously during the study period (through electronic reports) and reported monthly to clinic and pharmacy leadership. We will also conduct qualitative interviews with end-users before, during, and after intervention implementation to further assess the end user experience and for quality improvement. Patient and provider surveys will be conducted post-implementation.

8.2 SAFETY AND OTHER ASSESSMENTS

The CDS tool uses patient information in the electronic medical records to apply evidence-based algorithms that identify eligible patients, compute cardiovascular risks, prioritize cardiovascular risk factors, alert to potential medication issues, provide treatment recommendations, and promote adherence assessments and action plans. The study team has extensive experience in health services research and clinical research with human subjects, with procedures to safeguard privacy and personal information. See section 10.1.3 below on a detailed description of procedures put in place to assess and ensure safety of patient information.

8.3 ADVERSE EVENTS AND SERIOUS ADVERSE EVENTS

8.3.1 DEFINITION OF ADVERSE EVENTS (AE)

AE means any untoward medical occurrence associated with the use of an intervention in humans, whether or not considered intervention-related[107,108].

8.3.2 DEFINITION OF SERIOUS ADVERSE EVENTS (SAE)

An AE or suspected adverse reaction is considered "serious" if, in the view of either the investigator or sponsor, it results in any of the following outcomes: death, a life-threatening adverse event, inpatient hospitalization or prolongation of existing hospitalization, a persistent or significant incapacity or substantial disruption of the ability to conduct normal life functions, or a congenital anomaly/birth defect. Important medical events that may not result in death, be life-threatening, or require hospitalization may be considered serious when, based upon appropriate medical judgment, they may jeopardize the participant and may require medical or surgical intervention to prevent one of the outcomes listed in this definition. Examples of such medical events include severe hypoglycemia requiring intensive treatment in an emergency room or at home, elevated blood pressure or convulsions that do not result in inpatient hospitalization.

8.3.3 CLASSIFICATION OF AN ADVERSE EVENT

8.3.3.1 SEVERITY OF EVENT

For AEs not included in the protocol defined grading system, the following guidelines will be used to describe severity.

- **Mild** – Events require minimal or no treatment and do not interfere with the participant's daily activities.
- **Moderate** – Events result in a low level of inconvenience or concern with the therapeutic measures. Moderate events may cause some interference with functioning.
- **Severe** – Events interrupt a participant's usual daily activity and may require systemic drug therapy or other treatment. Severe events are usually potentially life-threatening or incapacitating. Of note, the term "severe" does not necessarily equate to "serious".

8.3.3.2 RELATIONSHIP TO STUDY INTERVENTION

All AEs must have their relationship to study intervention assessed by the clinician who examines and evaluates the participant based on temporal relationship and his/her clinical judgment. The degree of certainty about causality will be graded using the categories below.

- **Related** – The AE is known to occur with the study intervention, there is a reasonable possibility that the study intervention caused the AE, or there is a temporal relationship between the study intervention and event. Reasonable possibility means that there is evidence to suggest a causal relationship between the study intervention and the AE.
- **Not Related** – There is not a reasonable possibility that the administration of the study intervention caused the event, there is no temporal relationship between the study intervention and event onset, or an alternate etiology has been established.

8.3.3.3 EXPECTEDNESS

An AE will be considered unexpected if the nature, severity, or frequency of the event is not consistent with the risk information previously described for the study intervention.

8.3.4 TIME PERIOD AND FREQUENCY FOR EVENT ASSESSMENT AND FOLLOW-UP

With DSMB approval, this study will implement a passive surveillance strategy for potential AE's related to the study intervention. Data will be analyzed using model-predicted percentages of patients with safety conditions of interest for defined time periods during the intervention. Logistic regression models are used to generate predicted proportions by randomized treatment group controlling for age and baseline characteristics.

The occurrence of an AE or serious adverse event (SAE) may also come to the attention of project team members during patient and provider survey results and interviews with stake holders. These AEs not meeting the criteria for SAEs will be captured on the appropriate report forms. Information to be collected includes event description, time of onset, clinician's assessment of severity, relationship to study product (assessed only by those with the training and authority to make a diagnosis), and time of resolution/stabilization of the event. All AEs occurring while on study will be documented appropriately regardless of relationship. All AEs will be followed to adequate resolution.

Changes in the severity of an AE will be documented to allow an assessment of the duration of the event at each level of severity to be performed. AEs characterized as intermittent require documentation of onset and duration of each episode.

The project manager, in collaboration with the PI will record all reportable events with start dates occurring any time after inclusion into the study population until 7 (for non-serious AEs) or 30 days (for SAEs) after the last day of study participation. At each clinic visit, the project team member(s) will inquire about the occurrence of AE/SAEs since the last visit. Events will be followed for outcome information until resolution or stabilization.

8.3.5 ADVERSE EVENT REPORTING

According to the HealthPartners Institute IRB policies, investigators must report the specified adverse events or issues to the IRB as soon as possible, but in no event later than 10 working days after the investigator first learns of the event. Investigators or the project team must report possible problems or issues with the research to the IRB Office in writing using the Unanticipated Problem/Serious Adverse Event Form. The written report should contain the following: Detailed information about the event or issue, including relevant dates; Any corrective and preventative actions, planned or already taken, to ensure that the issue or problem is corrected and will not occur again; An assessment of whether any subjects or others were placed at risk as a result of the event or suffered any harm (e.g., physical, social, financial, legal or psychological) and any plan to address these consequences.

8.3.6 SERIOUS ADVERSE EVENT REPORTING

The study investigator shall complete an Unanticipated Adverse Device Effect Form and submit to the study sponsor and to the reviewing Institutional Review Board (IRB) as soon as possible, but in no event later than 10 working days after the investigator first learns of the effect.

8.3.7 REPORTING EVENTS TO PARTICIPANTS

Participating clinics will be informed of AEs and SAEs through communication with the clinic and healthcare group leadership and providers. Although unlikely, if specific patients are affected or at risk, they will be contacted through their routine patient communication channels and through their providers.

8.3.8 EVENTS OF SPECIAL INTEREST

Not-Applicable

8.3.9 REPORTING OF PREGNANCY

Pregnancy is an exclusion criteria for the study. However, reporting is not warranted as there is no direct harm anticipated from the study.

8.4 UNANTICIPATED PROBLEMS

8.4.1 DEFINITION OF UNANTICIPATED PROBLEMS (UP)

The Office for Human Research Protections (OHRP) considers unanticipated problems involving risks to participants or others to include, in general, any incident, experience, or outcome that meets ALL of the following criteria:

- Unexpected in terms of nature, severity, or frequency given (a) the research procedures that are described in the protocol-related documents, such as the Institutional Review Board (IRB)-approved research protocol and informed consent document; and (b) the characteristics of the participant population being studied;
- Related or possibly related to participation in the research ("possibly related" means there is a reasonable possibility that the incident, experience, or outcome may have been caused by the procedures involved in the research); and
- Suggests that the research places participants or others at a greater risk of harm (including physical, psychological, economic, or social harm) than was previously known or recognized.

8.4.2 UNANTICIPATED PROBLEM REPORTING

The investigator will report unanticipated problems (UPs) to the reviewing Institutional Review Board (IRB) and to HealthPartners Institute[108]. The UP report will include the following information:

- Protocol identifying information: protocol title and number, PI's name, and the IRB project number;
- A detailed description of the event, incident, experience, or outcome;
- An explanation of the basis for determining that the event, incident, experience, or outcome represents an UP;
- A description of any changes to the protocol or other corrective actions that have been taken or are proposed in response to the UP.

To satisfy the requirement for prompt reporting, UPs will be reported using the following timeline:

- UPs that are serious adverse events (SAEs) will be reported to the IRB and to the study sponsor within 5 days of the investigator becoming aware of the event.

- Any other UP will be reported to the IRB and the study sponsor within 10 working days of the investigator becoming aware of the problem.
- All UPs should be reported to appropriate institutional officials (as required by an institution's written reporting procedures), the supporting agency head (or designee), and the Office for Human Research Protections (OHRP) within 1 month of the IRB's receipt of the report of the problem from the investigator.

8.4.3 REPORTING UNANTICIPATED PROBLEMS TO PARTICIPANTS

Participating clinics will be informed of UPs through communication with the clinic and healthcare group leadership and providers. Although unlikely, if specific patients are affected or at risk, they will be contacted through their routine patient communication channels and through their providers.

9 STATISTICAL CONSIDERATIONS

9.1 STATISTICAL HYPOTHESES

- Primary Efficacy Endpoint(s):

For patients 18 to 75 years old not at goal for BP, A1C, or statin use and with medication nonadherence, those randomly assigned to the ReachOut Adherence Intervention will have improved adherence and clinical outcomes compared to those randomly assigned to control.
Hypothesis 1.1 Relative to those assigned to control, eligible adults with uncontrolled BP and baseline nonadherence to one or more BP medications who are assigned to intervention will be more likely to achieve (a) an e-PDC ≥80% for all BP medications and (b) a reduction in systolic BP in the 12-month period after the index visit.
Hypothesis 1.2 Relative to those assigned to control, eligible adults with diabetes and A1C ≥8% and baseline nonadherence to a non-insulin glycemic medication who are assigned to intervention will be more likely to achieve (a) e-PDC ≥80% for all non-insulin glycemic medications and (b) a reduction in A1C values in the 12-month period after the index visit.
Hypothesis 1.3 Relative to those assigned to control, eligible adults with inadequately managed lipid disorders with baseline non-adherence to a statin who are assigned to intervention will be more likely to achieve an e-PDC ≥80% for a statin in the 12-month period after the index visit.

- Secondary Efficacy Endpoint(s):

Assess the cost and cost-effectiveness of the ReachOut Adherence Intervention from the health system (payer) perspective through empirical analysis and microsimulation modeling of patient utilization and disease outcomes.
Hypothesis 2.1: Relative to those who are assigned to control, eligible study subjects who are assigned to intervention will have significantly higher overall health care costs in the 12-month period following the index visit, after controlling for demographics and baseline clinical risk factors.
Hypothesis 2.2: Relative to those who are assigned to control, eligible study subjects who are assigned to intervention will have significantly lower overall resource use and fewer major CV and diabetes-related events in a 30-year post-intervention simulated analysis using cost-effectiveness microsimulation modeling methods.

9.2 SAMPLE SIZE DETERMINATION

H1.1 (BP): Based on preliminary data collected in our study clinics between August 6, 2019 and December 23, 2019, we anticipate that 2,132 patients will meet study eligibility criteria of having at least one antihypertensive medication with e-PDC <80% over a 6-month enrollment period. For power analysis, we adjusted this estimate by half (N=1,066) to conservatively account for a potential continuation of the current decrease in office visit secondary to clinic, visit and provider changes as part of COVID-19 pandemic response. Further assuming a two-sided $\alpha=0.05$, equal comparison groups (1:1 intervention/control), and a probability of outcome (adherence) in the control group of 50%, the analysis for H1.1a will have 80% power to detect a difference in the proportion of patients with 12-month e-PDC ≥80% of 8.7% or more (e.g., 50.0% in control patients vs. 58.7% in intervention patients). For pre-post analyses of SBP outcomes, we estimate that to detect a clinically meaningful difference in mean 12-month SBP of 3 mmHg, assuming 80% power, $\alpha=0.05$, SD=15.8, 1:1 intervention/control, and $\rho=0.5$ (correlation of repeated measures), a sample size of N=872 patients will be required. Given the projections described above (N=1,066), we expect sufficient enrollment to meet this target sample size.

H1.2 (Glycaemia): From preliminary data we estimate that 1,664 patients will meet study eligibility criteria due to having at least one non-insulin glucose medication with e-PDC <80% and having a most recent A1C ≥8%. Adjusting this estimate by half (as above) we anticipate N=832. Assuming a two-sided $\alpha=0.05$, equal comparison groups (1:1 intervention/control), and a probability of outcome (adherence) in the control group of 50%, the analysis for H1.2a will have 80% power to detect a difference in the proportion of patients with a 12-month e-PDC ≥80% of 9.9% (e.g., 50.0% in control patients vs. 59.9% in intervention patients). For pre-post analyses of A1C outcomes, assuming $\alpha=0.05$, SD=1.7, 1:1 intervention/control, and $\rho=0.5$ (correlation of repeated measures), and N=832, we expect 80% power to detect a clinically meaningful difference in A1C of 0.3% (e.g., 9.7% in control patients vs. 9.4% in intervention patients).

H1.3 (Statin use): From preliminary data we estimate that 3,388 patients will meet study eligibility criteria due to having a statin-specific e-PDC <80%. Adjusting this estimate by half we anticipate N=1,694. Assuming a two-sided $\alpha=0.05$, 1:1 intervention/control, and a probability of outcome (adherence) of 50%, the analysis for H1.3 will have 80% power to detect a difference in the proportion of patients with a 12-month e-PDC ≥80% for statin medications of 6.9% (e.g., 50.0% in control patients vs. 56.9% in intervention patients).

9.3 POPULATIONS FOR ANALYSES

The **analysis population** is limited to subjects who meet intervention eligibility requirements at an index visit. We will use data from the Wizard algorithm runs outside the encounter at 6 and 12 months post-index visit to analyze the impact of the intervention on adherence (e-PDC) and clinical outcomes (A1C, BP, statin use). The cost analysis is limited to the subset of these patients who have HealthPartners insurance for at least 11 of 12 months after the index visit.

9.4 STATISTICAL ANALYSES

9.4.1 GENERAL APPROACH

The primary predictor is the study arm to which a patient is randomized. This variable is a one-degree-of-freedom contrast with the control group as the reference category and adherence intervention as the other category. Patient and provider characteristics will be documented in order to assess the extent to which results apply to subgroups of patients. Patient characteristics obtained from the EMR include demographics, health care payer, and comorbid conditions. We will have complete information for physician characteristics, including age, years since graduation, sex, full- or part-time work status, and physician or allied provider (e.g., nurse practitioner). Pharmacist characteristics will be collected by survey.

9.4.2 ANALYSIS OF THE PRIMARY EFFICACY ENDPOINT(S)

Definitions of Dependent Variables (Aim 1): The primary dependent variables are described in Table 3 below.

**Table 2. Description of Dependent Variables Used in Primary and Secondary Analysis.**			
**Variable**	**Description**	**Data Source**	**Classification**
PDC provided in the EMR **(H1.1a, H1.2a, H1.3)**	For each condition, achievement of e-PDC ≥80% for all medication classes for that particular condition (diabetes, hypertension, statin indication) in the 12 months following index date.	EMR	Binary
BP reduction (mmHg) **(H1.1b)**	Mean change in SBP from the last BP within the 12 months prior to the index visit, to the last BP value within 12 months after the index visit.	EMR vitals	Continuous
Change in A1C value **(H1.2b)**	Change in A1C value from the last lab test within the 12 months prior to the index visit, to the last lab test within 12 months after the index visit.	EMR procedure results	Continuous

Drug-specific PDCs are extracted from Epic EMR. Key secondary dependent variables (also defined in Table 3) include (a) total 10-year ACC/AHA CV risk, and (b) other objective measures of medications adherence such as PDC calculated using claims data, and (c) survey assessed patient self-reported adherence using a validated instrument.[110] We will assess additional dependent variables through a patient survey including barriers to adherence,[111] shared decision making,[112] health care satisfaction,[113] and provider and pharmacist knowledge, attitudes, beliefs, and satisfaction with the CDS.

Analysis Plan for Aim 1 (Adherence and Outcome Hypotheses): Hypotheses H1.1a, H1.2a, H1.3 posit that patients assigned to the adherence intervention will be more likely than patients assigned to usual care to have high medication adherence (e-PDC ≥80%) in the 12 months following the index visit for BP medications (H1.1a), glucose medications (H1.2a), and statin medications (H1.3). Generalized linear model (GLM) regression with a logit link and binomial error distribution will be used to test the effect of the intervention. The general form of the analytic primary model for testing medication adherence is: $logit(\Pr[PDC \geq 0.8]) = \beta_0 + \beta_1(Intervention) + \beta_c(Covariates)$

The dependent variable in each model (antihypertensive medications, glucose-lowering medications, statins) is a binary indicator of high medication adherence (e-PDC ≥80%) and is predicted by a binary indicator of adherence intervention assignment. We will screen and include patient characteristics in the model when they are unbalanced by treatment arm. In supplemental analyses, we will extend the GLM approach described above by including random intercepts to evaluate potential clustering effects at the level of clinic and/or provider.

H1 will be supported if the intervention group effect β_1 is significant and positive, supporting the prediction that clinical encounters with the adherence intervention are more effective than encounters without them for increasing medication adherence. Treatment group heterogeneity by patient sex, age group, race, insurance class, and comorbidity count will be assessed by including interaction terms consisting of products of patient variables and the intervention indicator.

Hypotheses H1.1b and H1.2b posit that patients assigned to the adherence intervention will be more likely than control patients to have reduced SBP (H1.1b) and reduced A1C (H1.2b) in the 12 months following the index visit.

$E(Y)_t = \beta_{00} + \beta_{10}(Intervention) + \beta_{01}(Time) + \beta_{11}(Intervention * Time) + \beta_{c0}(Covariates)$The analytic model will be a generalized linear model with an identity link and normal error distribution. The dependent variable *Y* (SBP for H1.1b and A1C for H1.2b) is predicted by a binary indicator of intervention assignment, time (pre-intervention vs. post), and the interaction of intervention assignment and time. We will screen and include patient characteristics in the model when they are unbalanced by treatment arm, and will evaluate the potential for clustering by clinic and/or provider.

A significant intervention group-by-time interaction term ($P<0.05$) and a pre-specified contrast indicating more reduction in SBP (H1.1b) or A1C (H1.2b) in the intervention than the control arm will support the prediction that encounters with the adherence intervention are more effective than encounters without it for improving BP and glycemic clinical outcomes.

Missing Data: All key analytic variables will be derived from EMR or health plan databases in which it is extremely rare for care-delivery information to be incompletely recorded. The primary endpoints for differential change in A1C and SBP by study arm will be analyzed using all available data at multiple time periods using direct likelihood-based ignorable methods obtained via generalized linear models, with no need to model the missing data process, and will yield valid inference when the outcome is missing at random.[114,115] For H1.1a, H1.2a, H1.3, if preliminary analyses determine that patient covariates are predictive of missing outcome data, we will consider using one or more of the following strategies. We will include these variables as covariates so that the model is conditioned on these characteristics. We will assume that missing values represent failed trials (e.g., a failure to realize one of the binary outcomes), resulting in conservative estimates of intervention effects. Finally, multiple imputation procedures may be employed to accurately estimate parameters across multiple imputed data sets.

9.4.3 ANALYSIS OF THE SECONDARY ENDPOINT(S)

Definition of Health Care Costs: Costs for this analysis include intervention costs and incremental medical care costs attributable to the intervention, defined from the health system perspective. Intervention costs include implementation, incentives to maintain high use rates, maintenance, and training but exclude research, development, and measurement costs.[116] Standard micro-cost accounting methods and nationally representative pricing of inputs will be used to measure the cost of the intervention. We include clinic payments to reimburse pharmacist time and resources for conducting active outreach. Medical care costs include the cost of all prescribed medications and clinic services—including laboratory, MTM pharmacist, educator, and physician services—incurred in the 12-month pre- and post-index date periods by participants in each study group, as indicated by insurance claims and clinical encounter data. Emergency department visits and hospitalizations may be too infrequent in the study sample to accurately predict a population wide impact. To avoid substantially increasing variance without adding accuracy to the cost assessment, we will include emergency department and inpatient costs only if a significant difference in these is observed.

While market prices generally are a good estimate of the costs for medical services, the paid amount in this claims system is specific to HP at a particular time and may provide a biased view of costs between pre- and post-intervention periods. To address this, we will use Total Care Relative Resource Values™

(TCRRVs), which are a nationally standardized set of measures that have been endorsed by the National Quality Forum[117] and are derived from Centers for Medicare and Medicaid Services (CMS) relative value units (RVUs). TCRRVs extend CMS RVU measures to include utilization categories, such as laboratory services and medications, which do not have CMS RVU weights[118]. Specifically, TCCRVs defined during the midpoint of patient accrual and follow-up (e.g., 2020) will be used to convert claims to represent U.S. dollars.

Analytic Plan for Health care costs (12-month Utilization): Incremental medical costs will be estimated using standard health econometric methods. A generalized estimating equation (typically assuming a gamma distribution and log link function) will be used to estimate costs by study arm while controlling for demographics and baseline clinical risk factors.[119-121] The marginal effect of being assigned to an intervention clinic will provide an estimate of the incremental medical cost associated with the ReachOut adherence intervention.

Definition of Long-term Cost and Health Outcomes: Health outcomes include incidence, morbidity, and mortality of CV disease (including myocardial infarction, stroke, congestive heart failure, and angina pectoris) and microvascular complications due to diabetes (including blindness, ulcer, first amputation with no previous ulcer, first amputation with previous ulcer, second amputation, and renal failure). Medical costs corresponding with the incidence and ongoing management of these conditions are based on nationally representative data derived from the Medical Expenditure Panel Survey.[122]

Analytic Plan for Long-term Costs and Outcomes: The HealthPartners Institute ModelHealthTM: A cardiovascular disease (CVD) microsimulation model will be used to estimate long-term outcomes and cost-effectiveness from the health system perspective.[123] ModelHealth: CVD is an annual-cycle microsimulation model that, in its base configuration, is parameterized to estimate lifetime CV events and disease costs in persons representative of the U.S. population. The model has been previously used to conduct a decision analysis for the U.S. Preventive Services Task Force recommendation on aspirin and to evaluate improvements in hypertension, lipid, and lifestyle management.[124-129]

The study population will be simulated at the patient level by assigning the known characteristics of the adherence intervention and control groups to synthetic counterparts. Age, sex, race/ethnicity, BMI, systolic BP, cholesterol, smoking status, CV disease history, diabetes status, A1C, and medication use for persons in each study group at the end of the study follow-up period will be entered into the model baseline. Linear, exponential, logarithmic and, if appropriate, polynomial trends, will be fit to the observed intervention differences in medication adherence, CV risk factors, and A1C (identified by H1.1-H.3 and secondary analyses) and used to project future differential trends between the simulated ReachOut adherence intervention and control groups. Incidence of major CV events will be determined using risk equations developed specifically for the model using Framingham Heart Study data.[130,131]

Calculation and reporting of incremental cost-effectiveness ratios (ICERs) will follow the guidelines of the Panel on Cost-Effectiveness in Health and Medicine.[132] ICERs will be estimated with a 30-year analytic horizon in the primary analysis, and 10- and 20-year horizons will be considered in secondary analyses. Sources of uncertainty will be evaluated using standard sensitivity methods.[133]

9.4.4 SAFETY ANALYSES

Trends in potential safety concerns for subjects will also be monitored and analyzed as indicated by the Data Safety and Monitoring plan outlined in the safety oversight section below.

9.4.5 BASELINE DESCRIPTIVE STATISTICS

Data extracted will contain patient demographic information including age, sex and race.

9.4.6 PLANNED INTERIM ANALYSES

Not-applicable

9.4.7 SUB-GROUP ANALYSES

Assessment of heterogeneity of treatment utilization and effects based on pre-specified subgroups of patients will incorporate a factor (e.g. sex) and its interaction with treatment arm in mixed models. A small number of a priori subgroup variables will be tested and include patient sex, age group, race, insurance class (Medicaid, Medicare, commercial), comorbidity count, and pharmacy characteristics.

9.4.8 TABULATION OF INDIVIDUAL PARTICIPANT DATA

Individual participant data will not be listed by measure and time point.

9.4.9 EXPLORATORY ANALYSES

In tertiary/exploratory analysis, we evaluate the impact of the intervention on 10-year CV risk, other objective measures of medication adherence such as PDC, survey assessed patient self-reported adherence, and patient and provider experience and satisfaction as assessed by surveys and interviews. We will also explore differences in intervention use across patient subgroups based on age, gender, race and ethnicity and visit characteristics (such as type of visit, reason for visit, or provider). Treatment arm differences will be assessed using a general or generalized linear mixed-model approach as with the primary analysis, with link functions and error distributions determined by the distribution of each outcome.

Patient and provider experience, satisfaction, facilitators and barriers to implementation will be evaluated using patient and provider surveys and qualitative interviews. Throughout the development and intervention periods, qualitative interviews with key stakeholders which may include clinic and pharmacy leaders, PCPs, clinic staff, pharmacists and patients will be conducted to assess end user experience, enhance design and maximize buy-in. Qualitative interviews may be recorded, transcribed and analyzed ethnographically until thematic saturation is achieved.[134] Post-implementation, a pharmacist and PCP survey will assess measures of satisfaction, knowledge, use of adherence information, and shared decision-making behaviors.[135,136] Patient surveys will be conducted with a random sample of about 700 intervention and about 700 control patients. The surveys will be conducted in 2 phases, the first phase occurring within 2 to 4 weeks after index visit and the second phase 2 to 4 weeks after addition to the pharmacist registry to give ample time for the pharmacist outreach to be completed in the intervention subjects >=6 months after the index visit. The second survey sample will include only patients who responded to a question on the first survey consenting to be called again in 6 months for another survey. Surveys will be conducted in a sequential multimode design to simultaneously minimize costs and nonresponse error.[137,138] Surveys will include questions pertaining to self-reported adherence, barriers to adherence,[111] shared decision making,[112] satisfaction[113], and quality of provider communication.[139] The patient survey sample size assumes 50% response rates, consistent with those regularly achieved by HP Center for Evaluation and Survey Research, and is large enough to show significance based on results observed with these instruments in previous trials conducted by our research team and others. Existing scales with known psychometric properties will be used for surveys where available. Where no existing scales are available, questions will be developed using best practices to minimize measurement error.[139]

10 SUPPORTING DOCUMENTATION AND OPERATIONAL CONSIDERATIONS

10.1 REGULATORY, ETHICAL, AND STUDY OVERSIGHT CONSIDERATIONS

10.1.1 INFORMED CONSENT PROCESS

10.1.1.1 CONSENT/ASSENT AND OTHER INFORMATIONAL DOCUMENTS PROVIDED TO PARTICIPANTS

As with previous CDS studies we have conducted, a waiver of consent for the study population will be requested for the following reasons[108]:
a) All open treatment medication options identified by the intervention are U.S. Food and Drug Administration-approved and evidence-based, as specified in the regional and national clinical practice guidelines. No care is advocated that is not evidence-based.
Thus, the care conforms to current standards of care and ought not to represent any additional risk to patients beyond the routine risk that all patients assume whenever they have contact with the medical care system. The CDS system has been used to support diabetes, hypertension and lipid management in two previously completed randomized clinical trials with DSMB monitoring that revealed no safety concerns.
(b) The CDS identifies and gives safety warnings about drug-drug and drug condition that are potentially unsafe to the patient, which is of minimal risk to possible benefit to each patient at each visit during the study period.
(c) At clinic training sessions/communications and on printed decision support tools, we emphasize that it is inappropriate for a PCP to follow suggested treatment options without further checking the clinical status of a given patient. A disclaimer on the CDS says that treatment options are based only on electronically available data and are not intended to be a substitute for clinical judgment.
(d) It would be impractical to consent patients (due to large numbers of patients) and impossible to answer the primary research questions (due to selection effects related to consent) if written informed consent of all patients were required.
(e) Procedures are in place to opt out of pharmacist phone calls based on patient or provider preference.
(f) The intervention is supported by the care system's leadership, implemented with their collaboration and support, and embedded in the care process at the clinic and department level. Therefore, it would be accessible to all providers and pharmacists, with use of the tool indicating implied consent.

For the random sample of patients surveyed as part of the study, study information will be included in the survey invitation letter and survey introduction during the phone call, and implied consent will be assumed for survey responders. Informed consent will be obtained prior to any interviews.

In addition to the internal HealthPartners Institute policies, HIPAA itself makes specific provision for waiver of authorization to use protected health information (PHI) for research recruitment purposes under some specific conditions, all of which this study meets[140].

At HealthPartners, patients can opt out of use of their data for research. HealthPartners Institute keeps a record of the small number of individuals who have opted out to avoid use of their data or attempts to recruit them into studies. We will exclude patients from analysis who have declined permission for their medical records to be used in research.

10.1.1.2 CONSENT PROCEDURES AND DOCUMENTATION

Not-applicable.

10.1.2 STUDY DISCONTINUATION AND CLOSURE

This study may be temporarily suspended or prematurely terminated if there is sufficient reasonable cause. Written notification, documenting the reason for study suspension or termination, will be provided by the suspending or terminating party to participating clinics, investigators, study team members and the funding agency. If the study is prematurely terminated or suspended, the Principal Investigator (PI) will promptly inform participating clinics, the Institutional Review Board (IRB), and sponsor and will provide the reason(s) for the termination or suspension.

Circumstances that may warrant termination or suspension include, but are not limited to:

- Determination of unexpected, significant, or unacceptable risk to participants
- Demonstration of efficacy that would warrant stopping
- Insufficient compliance to protocol requirements
- Data that are not sufficiently complete and/or evaluable
- Determination that the primary endpoint has been met
- Determination of futility

Study may resume once concerns about safety, protocol compliance, and data quality are addressed, and satisfy the sponsor and IRB.

10.1.3 CONFIDENTIALITY AND PRIVACY

In order to ensure patient confidentiality, we need to print the name of the patient on the CDS tools that are printed and given to the patient and their PCP at the time of a clinic visit. Because more than one nurse-PCP team may use the same printer, names are required to avoid mixing up printed materials and giving PHI to the wrong patient. It is also desirable to have each patient's name on this sheet of paper to ensure the patient knows that the information on the paper is personalized and related to his or her own health state. The technical aspects of this system are also relevant. The CDS system includes a Web service that applies a complex set of evidence-based algorithms and requires the patient name to create the materials that are subsequently displayed and printed within the EMR. Several measures are in place to ensure security of PHI: (1) Data transfer to and from the EMR, the Web service (at HealthPartners Institute), and the Web display (accessed at HealthPartners clinics and pharmacies and Goodrich pharmacies) uses a Simple Object Access Protocol (SOAP) with Secure Sockets Layer (SSL) encryption over Hypertext Transfer Protocol Secure (HTTPS); (2) The servers reside on an internal network protected by multiple firewalls; and (3) The Web server sits in the demilitarized zone (DMZ) so it can communicate with external systems. The rest of the processing and data storage occur on servers located inside the second firewall. No external system can access these servers directly, providing maximum security to the data that reside on these servers.

Although used for CDS display, patient names are scrubbed from the data nightly. The remaining data is retained in the operational data store for 2 weeks for immediate trouble shooting. Later, if needed for approved research, the data is moved to an analytic database with minimal access. With respect to the analytic databases, the following measures will be taken to protect PCPs and patients from the risk of breach of confidentiality: The patient name is not retained within the analytic database. A unique study ID code unrelated to the medical record number or other study subject-specific information will be assigned to each patient at the index visit (first visit in the intervention period). The study ID is used to link data from patient encounters over time and various data sources that are needed for analysis. A crosswalk table linking this code number to a PCP or patient name or medical record number will be destroyed within 12 months after completion of the linked databases needed to test study hypotheses.

In compliance with HIPAA regulations[140], no personally identifiable health information (PHI) will be shared outside of the affiliated covered entities (ACE) without obtaining a data use agreement or business associates agreement. HealthPartners Institute and HealthPartners are each one component of a larger organization defined as an ACE under HIPAA regulations. This allows researchers at the Institute to use HealthPartners medical records data for purposes such as those in this study in compliance with HIPAA regulations (i.e., following the concept of “minimally necessary” use of PHI).
The study team has extensive experience in health services research and clinical research with human subjects, with procedures to safeguard privacy and personal information. All study records are protected by:

- Locked storing all paper records in a secure location
- Use of untraceable study ID numbers instead of names wherever possible
- Password protection as well as firewalls
- Strong user login authentication on all electronic devices
- Physical security for all electronic devices containing personal information

Data will be retained in secure storage following the completion of the study in accordance with Minnesota and federal law. We guard against the potential for breach of subject confidentiality through a multi-layered system of data protection policies, processes, staff training, software safeguards and physical security measures for both paper and electronic data involved in research.
The following measures will be taken to protect subjects from the risk of breach of confidentiality:

- All data collected in the study will be identified by using a previously assigned arbitrary and unique subject identification number to each participant.
- A file containing a link between the study ID and individually identifying information will be maintained at by a programmer who is member of the study team through the conclusion of the study.
- A cross-walk table linking the study ID to a patient identity will be destroyed within 12 months after the linked databases needed to test study hypotheses are completed.
- All electronic study data will be maintained in a computerized database residing on a username- and password-protected file-server to which only the researchers involved in the study will have access.
- All study-related paper documents containing individually identifiable information will be maintained in locked file cabinets.

To protect the confidentiality of any HealthPartners employees participating in an interview, we will not allow anyone outside of the research team to know the identity of those interviewed. All of the protection to electronic data sources, described above, also apply to the interview audio recordings and transcripts.

Certificate of Confidentiality:

To further protect the privacy of study participants, a Certificate of Confidentiality will be issued by the National Institutes of Health (NIH). This certificate protects identifiable research information from forced disclosure. It allows the investigator and others who have access to research records to refuse to disclose identifying information on research participation in any civil, criminal, administrative, legislative, or other proceeding, whether at the federal, state, or local level. By protecting researchers and institutions from being compelled to disclose information that would identify research participants,

Certificates of Confidentiality help achieve the research objectives and promote participation in studies by helping assure confidentiality and privacy to participants.

10.1.4 FUTURE USE OF STORED SPECIMENS AND DATA

Data collected for this study will be analyzed and stored at the HealthPartners Institute. Data used by the CDS is stored at an operational data store for 2 weeks for immediate trouble shooting. Later, if needed for approved research, the data is moved to an analytic database with minimal access. After the study is completed, the de-identified, archived data will be transmitted to and stored at the Wizard Data Repository, for use by other researchers.

Sharing of study procedures and outcomes is an essential element of this research proposal. We are determined to ensure that data sharing occurs on a local, regional, and national level. Our plan includes the following:

Local: We will work closely with clinical and administrative leaders at HealthPartners and Park Nicollet to ensure that our adherence interventions will be well accepted and locally relevant. During the intervention and analysis phases of the project, we will continue to meet with these leaders to update them on our findings. If desired, once the intervention period is complete, we will activate the CDS at all HealthPartners and Park Nicollet primary care clinics in Minnesota and Wisconsin. Findings of our research will be presented locally at the HealthPartners Celebration of Research & Education and to local clinical and administrative leaders.

Regional: To ensure statewide availability and dissemination of results and interventions, Dr. Tom Kottke and Steve Simenson are influential regional and national opinion leaders in cardiovascular prevention and care and pharmacy management. In addition, findings of this research will be presented at regional conferences such as the Minnesota Health Services Research Conference and others. Results will also be communicated to the leaders at the Minnesota Department of Health and to other regional and statewide medical groups through the Institute for Clinical Systems Improvement, a regional shared-learning quality-improvement organization of which HealthPartners is a member.

National: Our main study findings will be presented at national meetings and published in peer-reviewed journals. Marsha Raebel, PharmD, former Senior Investigator, Kaiser Colorado, a well-recognized national expert in the adherence field, will help with national dissemination of our findings. In addition, our CDS tool is developed as a Web-based application to facilitate its adoption outside of HealthPartners, and the basic CDS has been implemented by three additional care systems. NIH research funding obtained in 2017 (R01HL133793) will expand the use of the basic CV CDS tool (without adherence information) to safety net clinics within the United States. The ‘safety net’ community health centers (CHCs) serving our nation’s most socioeconomically vulnerable populations are in great need of CDS that effectively supports CVD risk management, because CVD risks, incidence, and poor outcomes are substantially higher in CHC patients than the general population. We will offer support to operationalize the adherence-based enhancements to care systems that have implemented the basic CDS. If the funding agency desires, and if permitted under then-current law, at the conclusion of the funding period, we may provide a de-identified data set to the National Heart, Lung and Blood Institute at its written request for the use of other qualified researchers in the future.

10.1.5 KEY ROLES AND STUDY GOVERNANCE

Principal Investigator	Project Manager
JoAnn M. Sperl-Hillen, MD<br>*Senior Clinical Investigator*	*Lilian N. Chumba, MD, MScGH*<br>*Research Project Manager*
HealthPartners Institute	*HealthPartners Institute*
8170 33rd Ave S<br>*Mail Stop 23301A*<br>*Bloomington, MN 55425*	*8170 33rd Ave S*<br>*Mail Stop 23301A*<br>*Bloomington, MN 55425*
952-967-5009	*952-967-5279*
joann.m.sperlhillen@healthpartners.com	*lilian.n.chumba@healthpartners.com*
Other Team Members	
Co-Investigators	*Patrick O'Connor, Caitlin Frail, Pamala Pawloski*
Statistics and Data Management	*Jeffrey Anderson*
Health Economics	*Steve Dehmer*
Application Development	*Deepika Appana*

Consultants:
The project includes a strong team of external consultants, including nationally recognized experts in medication adherence (Marsha Raebel, PharmD, retired Senior Investigator, Kaiser Permanente Colorado) and retail pharmacy (Steve Simenson, President of Goodrich Pharmacy)

Steering group:
The study team has a steering group that meets regularly as needed. It includes the two consultants above as well as internal health plan and medical group leaders.

10.1.6 SAFETY OVERSIGHT

Safety oversight will be under the direction of a Data and Safety Monitoring Board (DSMB) composed of individuals with the appropriate expertise, including expertise in clinical trials methodology and the clinical domains addressed in the proposed research. The member board includes: Dr. Catherine Benziger, the Medical Director of Heart and Vascular research for Essentia Health, a Cardiologist in the Essentia Health Heart and Vascular Center, and an adjunct clinical professor at the University of Minnesota Medical School; Dr. Barry Blumenfeld, a Senior Physician Informaticist at RTI International with more than 25 years of experience in clinical informatics and executive leadership in health care information systems design, development, implementation, and optimization; Dr. Julian Wolfson, Ph.D, an Associate Professor, Division of Biostatistics, School of Public Health, University of Minnesota with more than 10 years' experience in biostatistics including clinical trials; and Dr. David Smith, PharmD, a PhD holder in Pharmaceutical Outcomes Research and Policy working as a Distinguished Investigator at the Center for Health Research in Kaiser Permanente Northwest. This well rounded board brings expertise in CV disease and diabetes, information technology, and statistical expertise. In addition, a representative of the funding agency, NHLBI, will be invited to participate via conference call at all formal DSMB meetings. The PI will participate in the DSMB meetings in a limited way, as recommended in NIH policy. The DSMB will meet at least semiannually to assess safety and efficacy data on each arm of the study. The DSMB will convene within 3 months of the intervention start and will meet twice annually throughout the intervention period. The DMSB will operate under the rules of an approved charter that will be written and reviewed at the organizational meeting of the DSMB. A special focus of interest will be the safety of patients exposed to the study intervention. With regard to the basic CDS (CDS without the adherence information), similar CDS has been provided to PCPs in two previous NIH-

funded projects by our research team (R01DK068314 and R01HL102144), and extensive examination of clinical data by those DSMBs has thus far failed to detect any adverse effects of these interventions. The ReachOut Adherence intervention provides point-of-care CDS that provides medication adherence scores and evidence-based treatment suggestions to PCPs, pharmacists, and adults aged 18-75 years with suboptimal A1C, BP, or lipids consistent with national guideline recommendations that have been further vetted by clinical leaders at HPMG and Goodrich pharmacies and outside consultants who have agreed to participate in this project. CDS recommendations provided as part of the ReachOut Adherence intervention are designed to support clinicians' decision-making, not to override clinical judgment. Adverse events information will be collected in accordance with DSMB recommendations by the study coordinator and recorded on standard forms based on those used in other trials (R01HL102144). Consistent with NIH and HealthPartners IRB policy, all adverse events will be promptly reported in writing to the NIH, the DSMB, and the HealthPartners IRB.

10.1.7 CLINICAL MONITORING

Not-applicable

10.1.8 QUALITY ASSURANCE AND QUALITY CONTROL

Quality control (QC) procedures will be implemented beginning with routine QC checks on the data capture system. Any missing data or data anomalies will be communicated to application and epic development teams for clarification/resolution.

The HealthPartners Institute will provide direct access to all trial related clinics, source data/documents, and reports for the purpose of monitoring and auditing by the sponsor, and inspection by local and regulatory authorities.

10.1.9 DATA HANDLING AND RECORD KEEPING

10.1.9.1 DATA COLLECTION AND MANAGEMENT RESPONSIBILITIES

i. Data sources

All necessary data to determine eligibility, implement and operate the intervention, test the study hypotheses and assess the impact of the intervention are derived from (a) electronic health records EHRs, (b) health plan or medical group administrative databases, (c) feedback received directly from patients and providers by email or through the CDS feedback tab/links (d) surveys of samples of eligible patients in both study arms, (e) surveys of participating PCPs and pharmacists, and (f) interview transcripts and field notes

ii. Process steps for data acquisition

At each patient encounter in an intervention or control clinic, the CDS Web service will use EPIC generated medication adherence scores to identify potential adherence issues. Clinical decision support generated using the adherence data will be presented only at intervention clinics. At the index visit (the first visit in the 12-month enrollment period that study inclusion and exclusion criteria are met), the patient is assigned a unique study identifier that is used to link patient encounter data over time. All index and subsequent encounter data for eligible patients, including medications and adherence calculations, are securely stored behind a firewall within a limited de-identified analysis dataset. No effort is made to prevent or discourage control clinics from using their usual methods to assess and address medication adherence.

All data collection conducted is blinded to randomization assignment. We will establish common study variable definitions drawn from national standards, HEDIS Measurement Sets, and definitions used in previous studies.[141] We will construct variable definitions and data extraction procedures for

demographics, enrollment characteristics, vital signs, pharmacy, inpatient and outpatient encounters, and diagnoses. We will develop conceptual and operational definitions and technical specifications for data elements without established definitions. Data from all sources, including encounter data, claims, and EMR data, will be restructured into a common format and data elements combined into uniform files. A unique identifier will link all person-level information. Data integrity will be assessed to ensure that observations are valid, reliable, and consistent. Each variable will be tested for completeness and out-of-range values. We will create Limited Data Sets that are consistent with human subjects' protection and HIPAA privacy regulations that will be kept at HP Institute, along with data dictionaries, coding manuals, and other documentation relevant to data collection or measurement issues. This resource may, subsequent to this study, be available to the funding agency or to other approved investigators in accordance with then-current regulations about data privacy and data use.

10.1.9.2 STUDY RECORDS RETENTION

Study documents will be retained for a minimum of 2 years after the last day of data collection.

10.1.10 PROTOCOL DEVIATIONS

The PI, Dr. JoAnn Sperl-Hillen, will lead weekly meetings of the investigator team to ensure that all project activities are conducted and completed in a timely fashion and exactly as specified in the study protocol. The team will identify and report any deviations within 5 working days of identification of the protocol deviation, or within 5 working days of the scheduled protocol-required activity. All deviations will be addressed in study source documents, reported to NHLBI and the Program Official. Protocol deviations will also be sent to the reviewing Institutional Review Board (IRB) per their policies. In addition, the programmers will meet weekly to deal with operational issues related to development, implementation, and maintenance of the intervention tools and with data and analysis issues throughout the project.

10.1.11 PUBLICATION AND DATA SHARING POLICY

This study will comply with the NIH Data Sharing Policy and Policy on the Dissemination of NIH-Funded Clinical Trial Information and the Clinical Trials Registration and Results Information Submission rule. As such, this trial will be registered at ClinicalTrials.gov, and results information from this trial will be submitted to ClinicalTrials.gov. In addition, every attempt will be made to publish results in peer-reviewed journals. Data from this study may be requested by other researchers 3 years after the completion of the primary endpoint or 2 years after the main paper of the trial has been published, whichever comes first, by contacting Dr. JoAnn Sperl-Hillen.

10.1.12 CONFLICT OF INTEREST POLICY

The independence of this study from any actual or perceived influence is critical. Therefore, any actual conflict of interest of persons who have a role in the design, conduct, analysis, publication, or any aspect of this trial will be disclosed and managed. Furthermore, persons who have a perceived conflict of interest will be required to have such conflicts managed in a way that is appropriate to their participation in the design and conduct of this trial. The study leadership in conjunction with the National Heart, Lung, Blood Institute has established policies and procedures for all study group members to disclose all conflicts of interest and will establish a mechanism for the management of all reported dualities of interest.

10.2 ADDITIONAL CONSIDERATIONS

Not applicable

10.3 ABBREVIATIONS

ACC/AHA	American College of Cardiology/American Heart Association
ACE	affiliated covered entities
AE(s)	Adverse Event(s)
ASCVD	Athersosclerotic cardiovascular Disease
AWPs	average wholesale prices
BP	Blood pressure
BPA	Best Practice Advisory
CDS	Clinical Decision Support
CFR	Code of Federal Regulations
CMS	Centers for Medicare & Medicaid Services
COC	Certificate of Confidentiality
CONSORT	Consolidated Standards of Reporting Trials
CPT4	Current Procedural Terminology version 4
CRF	Case Report Form
CV	Cardiovascular
DSMB	Data Safety Monitoring Board
DRE	Disease-Related Event
DRG(s)	diagnostic-related group(s)
EC	Ethics Committee
eCRF	Electronic Case Report Forms
EHR	Electronic Health Record
EMR	Electronic Medical Record
e-PDC	Epic provided Proportion of Days Covered
FDA	Food and Drug Administration
GCP	Good Clinical Practice
HIPAA	Health Insurance Portability and Accountability Act
HP	HealthPartners
HPMG	HealthPartners Medical Group
IB	Investigator's Brochure
ICD-10	10th revision of the International Statistical Classification of Diseases and Related Health Problems
ICERs	incremental cost-effectiveness ratios
ICH	International Conference on Harmonisation
ID	Identification
IMB	Information/Motivation/Beliefs
IRB	Institutional Review Board
LDL	Low-density lipoprotein
mg/dl	milligrams/deciliter
mm Hg	Millimeters of Mercury
MTM	Medication Therapy Management
NCT	National Clinical Trial
NIH	National Institutes of Health
OHRP	Office for Human Research Protections
PCMH	Patient-centered medical homes
PCP(s)	Primary Care Provider(s)
PDC	Proportion of Days Covered
PHI	personally identifiable health information
PI	Principal Investigator

PN	Park Nicollet
QC	Quality Control
RVU(s)	relative value unit(s)
SAE(s)	Serious Adverse Event(s)
SOA	Schedule of Activities
SOP	Standard Operating Procedure
UKPDS	United Kingdom Prospective Diabetes Study
UP	Unanticipated Problem
US	United States
VA	Veterans Affairs
WHO	World Health Organization

11 REFERENCES

References

1. Yeaw J, Benner JS, Walt JG, Sian S, Smith DB. Comparing adherence and persistence across 6 chronic medication classes. *J Manag Care Pharm.* 2009;15(9):728-740.

2. Cramer JA, Benedict A, Muszbek N, Keskinaslan A, Khan ZM. The significance of compliance and persistence in the treatment of diabetes, hypertension and dyslipidaemia: a review. *Int J Clin Pract.* 2008;62(1):76-87.

3. Osterberg L, Blaschke T. Adherence to medication. *The New England journal of medicine.* 2005;353(5):487-497.

4. Kennedy J, Tuleu I, Mackay K. Unfilled prescriptions of medicare beneficiaries: prevalence, reasons, and types of medicines prescribed. *J Manag Care Pharm.* 2008;14(6):553-560.

5. Pladevall M, Williams LK, Potts LA, Divine G, Xi H, Lafata JE. Clinical outcomes and adherence to medications measured by claims data in patients with diabetes. *Diabetes Care.* 2004;27(12):2800-2805.

6. Lage MJ, Hassan MK. The relationship between antipsychotic medication adherence and patient outcomes among individuals diagnosed with bipolar disorder: a retrospective study. *Ann Gen Psychiatry.* 2009;8:7.

7. Turner BJ, Hollenbeak C, Weiner MG, Ten Have T, Roberts C. Barriers to adherence and hypertension control in a racially diverse representative sample of elderly primary care patients. *Pharmacoepidemiology and drug safety.* 2009;18(8):672-681.

8. Caro JJ, Ishak KJ, Huybrechts KF, Raggio G, Naujoks C. The impact of compliance with osteoporosis therapy on fracture rates in actual practice. *Osteoporos Int.* 2004;15(12):1003-1008.

9. Sokol MC, McGuigan KA, Verbrugge RR, Epstein RS. Impact of medication adherence on hospitalization risk and healthcare cost. *Med Care.* 2005;43(6):521-530.

10. Noens L, van Lierde MA, De Bock R, et al. Prevalence, determinants, and outcomes of nonadherence to imatinib therapy in patients with chronic myeloid leukemia: the ADAGIO study. *Blood.* 2009;113(22):5401-5411.

11. Ganesan P, Sagar TG, Dubashi B, et al. Nonadherence to imatinib adversely affects event free survival in chronic phase chronic myeloid leukemia. *American journal of hematology.* 2011;86(6):471-474.

12. World Health Organization (WHO). Adherence to Long-term Therapies: Evidence for Action.

http://www.who.int/chp/knowledge/publications/adherence_report/en/index.html. Accessed March 2, 2017.

13. DiMatteo MR, Giordani PJ, Lepper HS, Croghan TW. Patient adherence and medical treatment outcomes: a meta-analysis. *Med Care.* 2002;40(9):794-811.

14. Stern M. Diabetes in Hispanic Americans. *National Diabetes Data Group, eds. Diabetes in America, 2nd ed.* Vol 95-1468. Bethesda, MD: National Institute of Diabetes and Digestive and Kidney Diseases; 1995:631-660.

15. Dragomir A, Cote R, White M, et al. Relationship between adherence level to statins, clinical issues and health-care costs in real-life clinical setting. *Value Health.* 2010;13(1):87-94.

16. Hiligsmann M, Rabenda V, Bruyere O, Reginster JY. The clinical and economic burden of non-adherence with oral bisphosphonates in osteoporotic patients. *Health Policy.* 2010;96(2):170-177.

17. Hiligsmann M, Rabenda V, Gathon HJ, Ethgen O, Reginster JY. Potential clinical and economic impact of nonadherence with osteoporosis medications. *Calcified tissue international.* 2010;86(3):202-210.

18. Heisler M, Choi H, Rosen AB, et al. Hospitalizations and deaths among adults with cardiovascular disease who underuse medications because of cost: a longitudinal analysis. *Med Care.* 2010;48(2):87-94.

19. Breitscheidel L, Stamenitis S, Dippel FW, Schoffski O. Economic impact of compliance to treatment with antidiabetes medication in type 2 diabetes mellitus: a review paper. *Journal of medical economics.* 2010;13(1):8-15.

20. Simpson SH, Eurich DT, Majumdar SR, et al. A meta-analysis of the association between adherence to drug therapy and mortality. *Bmj.* 2006;333(7557):15.

21. Rasmussen JN, Chong A, Alter DA. Relationship between adherence to evidence-based pharmacotherapy and long-term mortality after acute myocardial infarction. *Jama.* 2007;297(2):177-186.

22. Bryant LJ. Medicines adherence--evidence for any intervention is disappointing. *Journal of primary health care.* 2011;3(3):240-243.

23. Nease B, Miller S, Frazee SG, et al. *2010 Drug trend report: A market and behavior analysis.* St. Louis, MO: Express Scripts;2011.

24. Thinking outside the pillbox: A system-wide approach to improving patient medication adherence for chronic disease. *A NEHI Research Brief - August 2009* 2009; *http://www.nehi.net/writable/publication_files/file/pa_issue_brief_final.pdf*. Accessed March 2, 2017.

25. Senst BL, Achusim LE, Genest RP, et al. Practical approach to determining costs and frequency of adverse drug events in a health care network. *Am J Health Syst Pharm.* 2001;58(12):1126-1132.

26. McCarthy R. The price you pay for the drug not taken. *Business and health.* 1998;16(10):27-28, 30, 32-23.

27. McDonnell PJ, Jacobs MR. Hospital admissions resulting from preventable adverse drug reactions. *Ann Pharmacother.* 2002;36(9):1331-1336.

28. Sabate E. Adherence to long-term therapies: evidence for action, World Health Organization. 2003.

29. American College of Preventive Medicine. Medication Adherence Time Tool: Improving Health Outcomes. 2011; *http://www.acpm.org/?MedAdherTT_ClinRef*, May 18, 2016.

30. Roth MT, Ivey JL, Esserman DA, Crisp G, Kurz J, Weinberger M. Individualized medication assessment and planning: optimizing medication use in older adults in the primary care setting. *Pharmacotherapy.* 2013;33(8):787-797.

31. Avorn J, Monette J, Lacour A, et al. Persistence of use of lipid-lowering medications: a cross-national study. *Jama.* 1998;279(18):1458-1462.

32. DiMatteo MR, Giordani PJ, Lepper HS, Croghan TW. Patient adherence and medical treatment outcomes: a meta-analysis. *Medical Care.* 2002;40(9):794-811.

33. Cramer J. Partial medication compliance: the enigma in poor medical outcomes. *Am J Manag Care.* 1995;1:45-52.

34. Hughes D, Cowell W, Koncz T, Cramer J. Methods for integrating medication compliance and persistence in pharmacoeconomic evaluations. *Value Health.* 2007;10(6):498-509.

35. Zyczynski TM, Coyne KS. Hypertension and current issues in compliance and patient outcomes. *Curr Hypertens Rep.* 2000;2(6):510-514.

36. Ayoade A, Oladipo I. Evaluation of the correlation between self-report and electronic monitoring of adherence to hypertension therapy. *Blood pressure.* 2012;21(3):161-166.

37. Gonzalez JS, Schneider HE. Methodological issues in the assessment of diabetes treatment adherence. *Curr Diab Rep.* 2011;11(6):472-479.

38. Hess LM, Raebel MA, Conner DA, Malone DC. Measurement of adherence in pharmacy administrative databases: a proposal for standard definitions and preferred measures. *Ann Pharmacother.* 2006;40(7-8):1280-1288.

39. Raebel MA, Schmittdiel J, Karter AJ, Konieczny JL, Steiner JF. Standardizing terminology and definitions of medication adherence and persistence in research employing electronic databases. *Med Care.* 2013;51(8 Suppl 3):S11-21.

40. Vollmer WM, Xu M, Feldstein A, Smith D, Waterbury A, Rand C. Comparison of pharmacy-based measures of medication adherence. *BMC health services research.* 2012;12:155.

41. Epic corporation. How it works: How is Medication Adherence Determined? Epic 2018

42. Steiner JF. Rethinking adherence. *Annals of internal medicine.* 2012;157(8):580-585.

43. Hudon E, Beaulieu MD, Roberge D. Integration of the recommendations of the Canadian Task Force on Preventive Health Care: obstacles perceived by a group of family physicians. *Fam Pract.* 2004;21(1):11-17.

44. Tarn DM, Heritage J, Paterniti DA, Hays RD, Kravitz RL, Wenger NS. Physician communication when prescribing new medications. *Arch Intern Med.* 2006;166(17):1855-1862.

45. Solberg LI, Engebretson KI, Sperl-Hillen JM, O'Connor PJ, Hroscikoski MC, Crain AL. Ambulatory care quality measures for the 6 aims from administrative data. *Am J Med Qual.* 2006;21(5):310-316.

46. Stewart K, George J, Mc Namara KP, et al. A multifaceted pharmacist intervention to improve antihypertensive adherence: a cluster-randomized, controlled trial (HAPPy trial). *J Clin Pharm Ther.* 2014;39(5):527-534.

47. Tsuyuki RT, Houle SK, Charrois TL, et al. Randomized Trial of the Effect of Pharmacist Prescribing on Improving Blood Pressure in the Community: The Alberta Clinical Trial in Optimizing Hypertension (RxACTION). *Circulation.* 2015;132(2):93-100.

48. Giberson S, Yoder S, Lee MP. Improving Patient and Health System Outcomes through Advanced Pharmacy Practice. A Report to the U.S. Surgeon General. In: Pharmacist OotC, ed: U.S. Public Health Service; 2011.

49. Maynard RA, Wagner ME, Winkler SR, Montuoro JL. Assessment of student pharmacists' perceptions on participating in clinical services in the community pharmacy setting. *Curr Pharm Teach Learn.* 2011;3(2):123-136.

50. Margolis KL, Asche SE, Bergdall AR, et al. Effect of home blood pressure telemonitoring and pharmacist management on blood pressure control: a cluster randomized clinical trial. *Jama.* 2013;310(1):46-56.

51. Santschi V, Chiolero A, Colosimo AL, et al. Improving blood pressure control through pharmacist interventions: a meta-analysis of randomized controlled trials. *Journal of the American Heart Association.* 2014;3(2):e000718.

52. Bunting BA, Smith BH, Sutherland SE. The Asheville Project: clinical and economic outcomes of a community-based long-term medication therapy management program for hypertension and dyslipidemia. *J Am Pharm Assoc (2003).* 2008;48(1):23-31.

53. Reinke T. Medication therapy management program in N.C. saves $13 million. *Manag Care.* 2011;20(10):17-18.

54. Fera T, Bluml BM, Ellis WM. Diabetes Ten City Challenge: final economic and clinical results. *J Am Pharm Assoc (2003).* 2009;49(3):383-391.

55. Svarstad BL, Kotchen JM, Shireman TI, et al. Improving refill adherence and hypertension control in black patients: Wisconsin TEAM trial. *J Am Pharm Assoc (2003).* 2013;53(5):520-529.

56. Pringle JL, Boyer A, Conklin MH, McCullough JW, Aldridge A. The Pennsylvania Project: pharmacist intervention improved medication adherence and reduced health care costs. *Health Aff (Millwood).* 2014;33(8):1444-1452.

57. Joint Pharamacists Association. Medicare Program; Merit-Based Incentive Payment System and Alternative Payment Model Incentive under the Physician Fee Schedule, and Criteria for Physician-Focused Payment Models; Proposed Rule. In: Services CfMM, ed2016.

58. Kucukarslan SN, Hagan AM, Shimp LA, Gaither CA, Lewis NJ. Integrating medication therapy management in the primary care medical home: A review of randomized controlled trials. *Am J Health Syst Pharm.* 2011;68(4):335-345.

59. Centers for Medicare & Medicaid Services. Transforming Clinical Practice Initiative. 2016; *https://innovation.cms.gov/initiatives/Transforming-Clinical-Practices/*. Accessed March 2, 2017.

60. Newport F. Gallup Politics. Congress Retains Low Honesty Rating. 2012; *http://www.gallup.com/poll/159035/congress-retains-low-honesty-rating.aspx*. Accessed March 2, 2017.

61. Pharm D. Pharmacy Facts & Tools - Pharmacy Careers Summary. *http://www.uspharmd.com/student/facts_about_pharmacist/*. Accessed February 3, 2014.

62. Feldstein AC, Smith DH, Perrin N, et al. Improved therapeutic monitoring with several interventions: a randomized trial. *Arch Intern Med.* 2006;166(17):1848-1854.

63. Vora J. Access to a Health System's Electronic Medical Record. *Pharmacy Times.* 2015. *http://www.pharmacytimes.com/publications/health-system-edition/2015/september2015/access-to-a-health-systems-electronic-medical-record*. Accessed March 2, 2017.

64. American Pharmacists Association. Walgreens pharmacists begin using EHR's. 2014; *http://www.pharmacist.com/walgreens-pharmacists-begin-using-ehrs*. Accessed March 2 2017.

65. Downard S, Galt KA, Reel AB. Pharmacists' use of electronic health records: Silent leaders no more. *J Am Pharm Assoc (2003).* 2007;47(6):680.

66. Lau B, Overby CL, Wirtz HS, Devine EB. The association between use of a clinical decision support tool and adherence to monitoring for medication-laboratory guidelines in the ambulatory setting. *Appl Clin Inform.* 2013;4(4):476-498.

67. Bright TJ, Wong A, Dhurjati R, et al. Effect of clinical decision-support systems: a systematic review. *Annals of internal medicine.* 2012;157(1):29-43.

68. McKibbon KA, Lokker C, Handler SM, et al. The effectiveness of integrated health information technologies across the phases of medication management: a systematic review of randomized controlled trials. *Journal of the American Medical Informatics Association : JAMIA.* 2012;19(1):22-30.

69. Cleveringa FG, Gorter KJ, van den Donk M, van Gijsel J, Rutten GE. Computerized decision support systems in primary care for type 2 diabetes patients only improve patients' outcomes when combined with feedback on performance and case management: a systematic review. *Diabetes Technol Ther.* 2013;15(2):180-192.

70. Kawamoto K, Houlihan CA, Balas EA, Lobach DF. Improving clinical practice using clinical decision support systems: a systematic review of trials to identify features critical to success. *BMJ.* 2005;330(7494):765-.

71. Derose SF, Green K, Marrett E, et al. Automated outreach to increase primary adherence to cholesterol-lowering medications. *JAMA internal medicine.* 2013;173(1):38-43.

72. Dixon BE, Alzeer AH, Phillips EO, Marrero DG. Integration of Provider, Pharmacy, and Patient-Reported Data to Improve Medication Adherence for Type 2 Diabetes: A Controlled Before-After Pilot Study. *JMIR Med Inform.* 2016;4(1):e4.

73. Fisher WA, Fisher JD, Harman J. The Information-Motivation-Behavioral Skills Model: A General Social Psychological Approach to Understanding and Promoting Health Behavior. In: Suls J, Wallston KA, eds. *Social Psychological Foundations of Health and Illness*. Malden, MA, USA: Blackwell Publishing Ltd; 2003.

74. Goodell LS, Pierce MB, Amico KR, Ferris AM. Parental information, motivation, and behavioral skills correlate with child sweetened beverage consumption. *J Nutr Educ Behav.* 2012;44(3):240-245.

75. Gao J, Wang J, Zhu Y, Yu J. Validation of an information-motivation-behavioral skills model of self-care among Chinese adults with type 2 diabetes. *BMC Public Health.* 2013;13:100.

76. Schmaling KB, Blume AW, N A. A randomized controlled pilot study of motivational interviewing to change attitudes about adherence to medications for asthma. *J Clin Psychol Med Settings.* 2001;8:167-172.

77. Fisher JD, Fisher WA, Misovich SJ, Kimble DL, Malloy TE. Changing AIDS risk behavior: effects of an intervention emphasizing AIDS risk reduction information, motivation, and behavioral skills in a college student population. *Health Psychol.* 1996;15(2):114-123.

78. Carey MP, Maisto SA, Kalichman SC, Forsyth AD, Wright EM, Johnson BT. Enhancing motivation to reduce the risk of HIV infection for economically disadvantaged urban women. *Journal of consulting and clinical psychology.* 1997;65(4):531-541.

79. Bandura A. *Social Foundations of Thought and Action: A Social Cognitive Theory.* NJ: Prentic Hall; 1986.

80. Lazarus RS. Coping with the stress of illness. *WHO Reg Publ Eur Ser.* 1992;44:11-31.

81. Folkman S. Personal control and stress and coping processes: a theoretical analysis. *Journal of personality and social psychology.* 1984;46(4):839-852.

82. Prochaska JO, Velicer WF. The transtheoretical model of health behavior change. *Am J Health Promot.* 1997;12(1):38-48.

83. Newman S, Steed L, Mulligan K. Self-management interventions for chronic illness. *Lancet.* 2004;364(9444):1523-1537.

84. Adult Meducation: Improving Medication Adherence in Older Adults. Dimension 5: Patient-Related Factors. *http://www.adultmeducation.com/patientrelatedfactors_2.html*. Accessed May 13, 2016.

85. Gilmer TP, O'Connor PJ, Sperl-Hillen JM, et al. Cost-Effectiveness of an Electronic Medical Record Based Clinical Decision Support System. *Health services research.* 2012.

86. O'Connor PJ, Crain AL, Rush WA, Sperl-Hillen JM, Gutenkauf JJ, Duncan JE. Impact of an electronic medical record on diabetes quality of care. *Ann Fam Med.* 2005;3(4):300-306.

87. Roshanov PS, Fernandes N, Wilczynski JM, et al. Features of effective computerised clinical decision support systems: meta-regression of 162 randomised trials. *BMJ (Clinical research ed).* 2013;346:f657.

88. Rothman RL, DeWalt DA, Malone R, et al. Influence of patient literacy on the effectiveness of a primary care-based diabetes disease management program. *Journal of the American Medical Association.* 2004;292(14):1711-1716.

89. Dewalt DA, Berkman ND, Sheridan S, Lohr KN, Pignone MP. Literacy and health outcomes: a systematic review of the literature. *Journal of general internal medicine.* 2004;19(12):1228-1239.

90. O'Connor AM, Llewellyn-Thomas HA, Flood AB. Modifying unwarranted variations in health care: shared decision making using patient decision aids. *Health affairs (Project Hope).* 2004;Suppl Variation:VAR63-72.

91. Damschroder LJ, Aron DC, Keith RE, Kirsh SR, Alexander JA, Lowery JC. Fostering implementation of health services research findings into practice: a consolidated framework for advancing implementation science. *Implementation science : IS.* 2009;4:50.

92. Desai JR, Sperl-Hillen JM, O‘Connor PJ. Patient preferences in diabetes care: overcoming barriers using new strategies. *Journal of Comparative Effectiveness Research.* 2013;2(4):351-354.

93. Kharbanda EO, Nordin JD, Sinaiko AR, et al. TeenBP: Development and Piloting of an EHR-Linked Clinical Decision Support System to Improve Recognition of Hypertension in Adolescents. *EGEMS (Wash DC).* 2015;3(2):1142.

94. O'Connor P. Opportunities to Increase the Effectiveness of EHR-Based Diabetes Clinical Decision Support. *Appl Clin Inform.* 2011;2(3):350-354.

95. O'Connor PJ, Desai JR, Butler JC, Kharbanda EO, Sperl-Hillen JM. Current status and future prospects for electronic point-of-care clinical decision support in diabetes care. *Curr Diab Rep.* 2013;13(2):172-176.

96. O'Connor PJ, Sperl-Hillen JM, Rush WA, et al. Impact of Electronic Health Record Clinical Decision Support on Diabetes Care: A Randomized Trial. *Ann Fam Med.* 2011;9(1):12-21.

97. Sperl-Hillen JM, Averbeck B, Palattao K, Amundson J, Rush B, PJ OC. Outpatient EHR-Based Diabetes Clinical Decision Support That Works: Lessons Learned From Implementing Diabetes Wizard. *Diabetes Spectrum.* 2010;23(3):150-154.

98. Ruggiero L, Prochaska JO. Readiness for change: application of the transtheoretical model to diabetes. *Diabetes Spectrum.* 1993;6(1):21-60.

99. Ruggiero L, Rossi JS, Prochaska JO, et al. Smoking and diabetes: readiness for change and provider advice. *Addict Behav.* 1999;24(4):573-578.

100. Prochaska J, Velicer W, Rossi J, et al. Stages of change and decisional balance for 12 problem behaviors. *Health Psychol.* 1994;13(1):39-46.

101. Boyle R, O'Connor P, Pronk N, Tan A. Stages of change for physical activity, diet, and smoking among HMO members with chronic conditions. *Am J Health Promot.* 1998;12(3):170-175.

102. Peterson KA, Hughes M. Readiness to change and clinical success in a diabetes educational program. *J Am Board Fam Pract.* 2002;15(4):266-271.

103. Wubben DP, Vivian EM. Effects of pharmacist outpatient interventions on adults with diabetes mellitus: a systematic review. *Pharmacotherapy.* 2008;28(4):421-436.

104. Glasgow RE. RE-AIMing research for application: ways to improve evidence for family medicine. *Journal of the American Board of Family Medicine : JABFM.* 2006;19(1):11-19.

105. Damschroder LJ, Aron DC, Keith RE, Kirsh SR, Alexander JA, Lowery JC. Fostering implementation of health services research findings into practice: a consolidated framework for advancing implementation science. *Implementation science : IS.* 2009;4:50.

106. Wagner EH. Chronic disease management: what will it take to improve care for chronic illness? *Eff Clin Pract.* 1998;1(1):2-4.

107. Gliklich RE, Dreyer NA, Leavy MB, editors. Registries for Evaluating Patient Outcomes: A User's Guide [Internet]. 3rd edition. Rockville (MD): Agency for Healthcare Research and Quality (US); 2014 Apr. 12, Adverse Event Detection, Processing, and Reporting. Available from: *https://www.ncbi.nlm.nih.gov/books/NBK208615/*

108. Kelly McCormick. HealthPartners Institute IRB Policies and procedures. Available from https://sites.healthpartners.com/hp/InstiNet/irb/SiteAssets/SitePages/Home/IRB%20SOPs.pdf

109. Centers for Medicare & Medicaid Services. Medicare 2016 Part C & D Star Rating Technical Notes. *https://www.cms.gov/Medicare/Prescription-Drug-Coverage/PrescriptionDrugCovGenIn/Downloads/2016-Technical-Notes-Preview-1-v2015_08_05.pdf*. Accessed March 2, 2017.

110. Morisky DE, Green LW, Levine DM. Concurrent and predictive validity of a self-reported measure of medication adherence. *Med Care.* 1986;24(1):67-74.

111. Risser J, Jacobson TA, Kripalani S. Development and psychometric evaluation of the Self-efficacy for Appropriate Medication Use Scale (SEAMS) in low-literacy patients with chronic disease. *J Nurs Meas.* 2007;15(3):203-219.

112. O'Connor AM. Validation of a decisional conflict scale. *Med Decis Making.* 1995;15(1):25-30.

113. Agency for Healthcare Research and Quality. Preparing a Questionnaire Using the CAHPS Health Plan Survey 4.0. Rockville, MD. 2007.

114. Beunckens C, Molenberghs G, Kenward MG. Direct likelihood analysis versus simple forms of imputation for missing data in randomized clinical trials. *Clin Trials.* 2005;2(5):379-386.

115. Little RJA, Rubin DB. The Analysis of Social Science Data with Missing Values. *Sociological Methods Research.* 1989;18(2-3):292-326.

116. Gold M, Siegel J, Russell L, Weinstein M, eds. *Cost-Effectiveness in Health and Medicine.* New York: Oxford University Press; 1996.

117 National Quality Forum. Press release: NQF Endorses Resource Use Measures. January 31, 2012; http://www.qualityforum.org/News_And_Resources/Press_Releases/2012/NQF_Endorses_Resource_Use_Measures.aspx. Accessed June 12, 2018.

118. HealthPartners. Total Care Relative Resource Value (TCRRV™): A Measurement Approach to Achieve the Triple Aim. *HealthPartners White Paper.* September 21, 2017; https://www.healthpartners.com/ucm/groups/public/@hp/@public/documents/documents/cntrb_039627.pdf. Accessed June 12, 2018.

119. Blough DK, Madden CW, Hornbrook MC. Modeling risk using generalized linear models. *J Health Econ.* 1999;18(2):153-171.

120. Manning WG, Mullahy J. Estimating log models: to transform or not to transform? *J Health Econ.* 2001;20(4):461-494.

121. Kung Yee L, Zeger S. Longitudinal data analysis using generalized linear models. *Biometrika.* 1986;73(1):13-22.

122. Agency for Healthcare Research and Quality. Medical Expenditure Panel Survey. 2001-2010; *http://meps.ahrq.gov/mepsweb/*. Accessed March 2, 2017.

123. HealthPartners Institute for Education and Research. ModelHealth Microsimulation Models. 2015. *https://www.healthpartners.com/hprf/modelhealth/index.html*. Accessed March 2, 2017.

124. Dehmer SP, Maciosek MV, Flottemesch TJ, LaFrance AB, Whitlock EP. Aspirin for the Primary Prevention of Cardiovascular Disease and Colorectal Cancer: A Decision Analysis for the U.S. Preventive Services Task Force. *Annals of internal medicine.* 2016.

125. Dehmer SP, Maciosek MV, Flottemesch TJ. Aspirin Use to Prevent Cardiovascular Disease and Cancer: A Decision Analysis. *AHRQ Publication No. 15-05229-EF-1.* Rockville, MD: Agency for Healthcare Research and Quality: AHRQ; 2015.

126. Maciosek MV, LaFrance AB, Dehmer SP, et al. Updated priorities among effective clinical preventive services. *Annals of Fam Med.* 2017;15(1):14-22.

127. Dehmer SP, Maciosek MV, LaFrance AB, TJ F. Health Benefits and Cost-Effectiveness of Asymptomatic Screening for Hypertension and High Cholesterol and Aspirin Counseling for Primary Prevention. *Annal of Fam Med.* 2017;15(1):23-36.

128. Dehmer SP, Baker-Goering MM, Maciosek MV, et al. Modeled Health and Economic Impact of Team-Based Care for Hypertension. *Am J Prev Med.* 2016;50(5 Suppl 1):S34-44.

129. Dehmer SP. *The economics of a new health technology: an evaluation of the impact of statins on lifestyle behaviors*. Minneapolis, MN, University of Minnesota; 2012.

130. Gorelick PB, Mazzone T. Plasma lipids and stroke. *J Cardiovasc Risk.* 1999;6(4):217-221.

131. Tight blood pressure control and risk of macrovascular and microvascular complications in type 2 diabetes: UKPDS 38. UK Prospective Diabetes Study Group. *BMJ.* 1998;317(7160):703-713.

132. Gold M, Siegel J, Russell L, Weinstein M. *Cost Effectiveness in Health and Medicine.* Oxford University Press; 1996.

133. Briggs AH, Weinstein MC, Fenwick EA, Karnon J, Sculpher MJ, Paltiel AD. Model parameter estimation and uncertainty analysis: a report of the ISPOR-SMDM Modeling Good Research Practices Task Force Working Group-6. *Med Decis Making.* 2012;32(5):722-732.

134. Lewis, S. (2015). Qualitative Inquiry and Research Design: Choosing Among Five Approaches. *Health Promotion Practice*, *16*(4), 473–475. *https://doi.org/10.1177/1524839915580941*

135. Scholle SH, Pawlson LG, Solberg LI, et al. Measuring practice systems for chronic illness care: accuracy of self-reports from clinical personnel. *Joint Commission journal on quality and patient safety / Joint Commission Resources.* 2008;34(7):407-416.

136. Solberg LI, Crain AL, Tillema J, Scholle SH, Fontaine P, Whitebird R. Medical home transformation: a gradual process and a continuum of attainment. *Ann Fam Med.* 2013;11 Suppl 1:S108-114.

137. De Leeuw ED. To Mix or Not to Mix Data Collection Modes in Surveys. *Journal of Official Statistics.* 2005;21(2):233-255.

138. Dillman DA, Smyth JD, Christin LM. *Internet, Mail and Mixed-Mode Surveys: The Tailor Design Method.* Hoboken, NJ: Wiley & Sons; 2009.

139. Agency for Healthcare Research and Quality. CAHPS Clinician & Group Surveys *https://cahps.ahrq.gov/Surveys-Guidance/CG/index.html*. Accessed October 31, 2014.

140. HIPPA Privacy Rule and Its Impacts on Research http://privacyruleandresearch.nih.gov/pr_08.asp#8c

141. National Committee for Quality Assurance. HEDIS Technical Measurement Specifications. *http://www.ncqa.org/HEDISQualityMeasurement/HEDISMeasures/HEDIS2014.aspx*. Accessed October 31, 2014.
