# Supplementary material for: Clinical Decision Support and Cardiometabolic Medication Adherence: A Randomized Clinical Trial
Source: JAMA Netw Open. 2025 Jan 9;8(1):e2453745. doi: 10.1001/jamanetworkopen.2024.53745 (PMC11718557; doi:10.1001/jamanetworkopen.2024.53745)
Supplement: Supplement 3. — Data Sharing Statement [file jamanetwopen-e2453745-s003.pdf]

# Data Sharing Statement

O'Connor. Clinical Decision Support and Cardiometabolic Adherence. *JAMA Netw Open*. Published January 09, 2025. doi:10.1001/jamanetworkopen.2024.53745

**Data**
**Additional Information:** National Clinical Trial (NCT) Identified Number: NCT03748420
https://classic.clinicaltrials.gov/ct2/show/NCT03748420
**Data available:** Yes
**Data types:** Deidentified participant data
**How to access data:** Patrick.J.OConnor@HealthPartners.com
**When available:** With publication

**Supporting Documents**
**Document types:** None

**Additional Information**
**Who can access the data:** Qualified Investigators
**Types of analyses:** Data summaries congruent with current privacy regulations
**Mechanisms of data availability:** By contacting Patrick O'Connor
**Any additional restrictions:** Data from this study may be requested by other researchers 3 years after the completion of the primary endpoint or 2 years after the main paper of the trial has been published, whichever comes first
